# Evidence for Stabilizing Selection on Codon Usage in Chromosomal Rearrangements of *Drosophila pseudoobscura*

**DOI:** 10.1534/g3.114.014860

**Published:** 2014-10-17

**Authors:** Zachary L. Fuller, Gwilym D. Haynes, Dianhui Zhu, Matthew Batterton, Hsu Chao, Shannon Dugan, Mehwish Javaid, Joy C. Jayaseelan, Sandra Lee, Mingmei Li, Fiona Ongeri, Sulan Qi, Yi Han, Harshavardhan Doddapaneni, Stephen Richards, Stephen W. Schaeffer

**Affiliations:** * Department of Biology, The Pennsylvania State University, University Park, Pennsylvania 16802-5301; † Human Genome Sequencing Center, Baylor College of Medicine, Houston, Texas 77030

**Keywords:** codon bias, stabilizing selection, chromosomal inversions, recombination

## Abstract

There has been a renewed interest in investigating the role of stabilizing selection acting on genome-wide traits such as codon usage bias. Codon bias, when synonymous codons are used at unequal frequencies, occurs in a wide variety of taxa. Standard evolutionary models explain the maintenance of codon bias through a balance of genetic drift, mutation and weak purifying selection. The efficacy of selection is expected to be reduced in regions of suppressed recombination. Contrary to observations in *Drosophila melanogaster*, some recent studies have failed to detect a relationship between the recombination rate, intensity of selection acting at synonymous sites, and the magnitude of codon bias as predicted under these standard models. Here, we examined codon bias in 2798 protein coding loci on the third chromosome of *D. pseudoobscura* using whole-genome sequences of 47 individuals, representing five common third chromosome gene arrangements. Fine-scale recombination maps were constructed using more than 1 million segregating sites. As expected, recombination was demonstrated to be significantly suppressed between chromosome arrangements, allowing for a direct examination of the relationship between recombination, selection, and codon bias. As with other *Drosophila* species, we observe a strong mutational bias away from the most frequently used codons. We find the rate of synonymous and nonsynonymous polymorphism is variable between different amino acids. However, we do not observe a reduction in codon bias or the strength of selection in regions of suppressed recombination as expected. Instead, we find that the interaction between weak stabilizing selection and mutational bias likely plays a role in shaping the composition of synonymous codons across the third chromosome in *D. pseudoobscura*.

The majority of amino acids can be encoded by multiple or synonymous codons because the genetic code is degenerate. In the absence of mutation, drift, or selection, synonymous codons are expected to occur at equal frequencies for each amino acid (Hershberg and Petrov 2008). In a wide variety of species, ranging from viruses to mammals, synonymous codons are used at different frequencies, a phenomenon known as codon bias (Ikemura 1981; Akashi 1994; Lynn *et al.* 2002; Novella *et al.* 2004; Parmley *et al.* 2006). Explanations for codon bias have traditionally fallen into two classes. The neutral explanation hypothesizes that bias exists because of nonrandom mutation patterns and genetic drift. Alternatively, weak directional selection has been hypothesized to generate codon bias through differences in translational accuracy and/or efficiency of an mRNA in the production of their encoded proteins (Akashi 1994). Standard evolutionary models often incorporate weak purifying selection, together with the effects of mutation and drift, to explain the phenomenon of codon usage bias (Li 1987; Bulmer 1991; McVean and Charlesworth 1999). Recently, however, there has been a renewed interest in the possible role of stabilizing selection on quantitative traits such as codon usage bias that extend from the neutral theory models of Kimura (1981, 1983; Charlesworth 2013).

The most frequently used synonymous codon is known as the “major” codon, whereas those used less frequently are known as “minor” codons. Mutations changing a minor codon to a major codon, hence increasing the frequency of major codons within a gene, are known as “preferred” changes. Alternatively, “unpreferred” mutations decrease the frequency of major codons by changing a major codon to a minor one. The magnitude of codon bias varies considerably between genes within a species and is found to be positively correlated with the relative level of gene expression (Sharp and Li 1987). It has been shown that artificially changing the numbers of major codons in a gene can alter the level of expression (Carlini and Stephan 2003; Hense *et al.* 2010).

The effective population size, *N_e_*, is thought to be a major factor contributing to the efficacy of natural selection (Charlesworth 2009). For many traits, selection can act more strongly and more efficiently in larger populations, as has been demonstrated in several species of *Drosophila* (Andolfatto *et al.* 2011). However, recent theoretical models of stabilizing selection predict that if sufficient mutational bias exists to alter the population mean of a quantitative trait away from its optimum value, the intensity of selection is nearly independent of *N_e_* (Charlesworth 2013). Standard models of selection on codon usage predict that the intensity of selection scales with *N_e_* (Li 1987; Bulmer 1991).Within a species, *N_e_* can vary within the genome of a species due to differences in recombination rates across chromosomes and the effects of background selection. Regions of the genome where recombination is suppressed have a lower *N_e_* than genomic regions where recombination is unimpeded (Comeron *et al.* 1999; Comeron and Kreitman 2002; Gossmann *et al.* 2011). If codon usage is sensitive to *N_e_* or the rate of recombination, one would expect to observe differences in the frequency of major codons in regions of suppressed recombination, where *N_e_* is reduced, relative to regions where recombination is not inhibited.

Suppressed recombination is expected to reduce the efficacy of natural selection due to linkage disequilibrium among loci under selection, an effect known as Hill-Robertson interference (HRI; Hill and Robertson 1966; McVean and Charlesworth 2000). Consistent with this expectation, several studies in *D. melanogaster* and various other *Drosophila* species have demonstrated that the level of codon bias is drastically reduced in regions of limited recombination that generate up to an approximate ten-fold reduction in *N_e_* (Bachtrog 2003, 2007; Haddrill *et al.* 2007; Betancourt *et al.* 2009; Arguello *et al.* 2010). However, recent work has highlighted cases in which the observed patterns of codon usage and base composition contradict predictions based on Hill-Robertson interference. Campos *et al.* (2013) estimated the effective population size of the X chromosome to be approximately three-quarters of the autosomes in a Rwandan population of *D. melanogaster* yet still observed significantly greater levels of codon bias on the X. This pattern of codon usage cannot be explained by mutational bias or biased gene conversion either, and is instead suggestive of either stronger selection on the X or a departure from traditional models of directional selection on synonymous sites. Additionally, Kliman (2014) did not find a significant association between recombination and the strength of selection on codon usage on the second chromosome of *D. pseudoobscura*. These recent results emphasize the complex interaction of mutation, recombination and natural selection in determining the pattern of base composition and codon usage in a genome, as well as suggest a possible role of stabilizing selection.

The third chromosome of *D. pseudoobscura* is highly polymorphic for different gene arrangements that were generated through a series of >30 overlapping paracentric inversion mutations. This gene arrangement polymorphism has been stable in natural populations for more than 70 years (Figure 1A; Dobzhansky and Sturtevant 1938). Frequencies of particular chromosome arrangements form a classical cline across the species range in the American southwest despite the predicted effects of extensive migration, indicating the potential adaptive nature of the inversion mutations (Figure 1B; Schaeffer *et al.* 2003; Wallace *et al.* 2011, 2013). Several molecular studies have estimated the population scaled migration rate, *N_e_m*, to be greater than 1, which is sufficient to homogenize gene arrangement frequencies (Riley *et al.* 1989; Schaeffer and Miller 1992; Kovacevic and Schaeffer 2000; Schaeffer *et al.* 2003). An unrooted network representing the phylogenetic relationship (Figure 1C) between the most common gene arrangements has been soundly established through a long history of cytological and molecular studies dating back to the pioneering work of Dobzhansky and Sturtevant (1938). One arrangement was arbitrarily chosen as the Standard (ST) chromosome, and each new arrangement discovered was named after the location where it was first collected. These include the Arrowhead (AR), Pikes Peak (PP), Tree Line (TL), and Chiricahua (CH) arrangements. The inferred ancestral arrangement at the center of the phylogeny, Hypothetical (HY), has never been observed in nature. Distances vary between inversion breakpoints for each arrangement (Table 1), ranging from the ST→PP inversion, which spans more than 12 Mb, to the HY→ST inversion, which spans just less than 3 Mb. The closest breakpoint to either the centromere or telomere is within 2.5 Mb (S. W. Schaeffer, unpublished data). Recombination is not hypothesized to be suppressed between heterokaryotypes in these homosequential regions.

**Figure 1:**
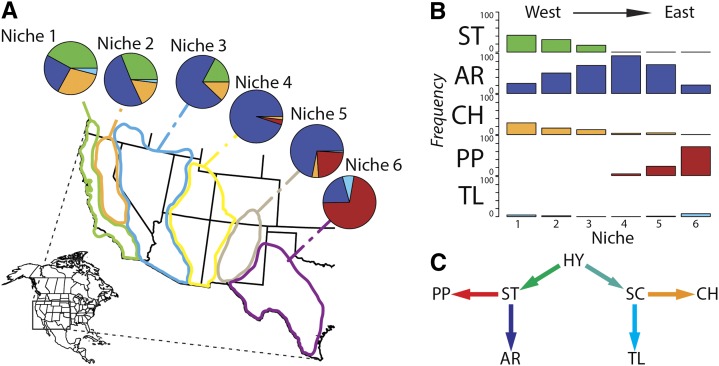
The third chromosome inversion polymorphism of *D. pseudoobscura*. (A) The frequency and spatial distribution of *D. pseudoobscura* third chromosome gene arrangements (after Schaeffer 2008). Arrowhead (blue) has the largest distribution and is observed in all defined niches across the southwestern United States. (B) The relative frequency of arrangements in niches moving west to east. (C) The inversion phylogeny of *D. pseudoobscura* third chromosome arrangements. The Hypothetical (HY) arrangement is ancestral; however, it has never been observed in nature. Tree Line (TL) and Chiricahua (CH) are derived from the Santa Cruz (SC) arrangement, whereas Pikes Peak (PP) and Arrowhead (AR) are derived from the Standard (ST) arrangement. Each colored arrow represents an inversion event.

**Table 1:** Approximate location of inversion breakpoints on the third chromosome

Breakpoint^a^	Cytological	Left Gene	Right Gene	Reference Location
pSTPP	65B|65C	GA13487	GA13475	2,496,966–2,502,362
pHYSC	68B|68C	GA21475	GA29564	6,810,759–6,819,459
pSTAR	70A|76B	GA24334	GA19155	8,899,726–8,902,522
pHYST	76B|76A	GA22082	GA20777	9,156,632–9,161,151
dSTPP	76B|76A	GA22802	GA20777	9,156,632–9,161,151
pSCTL	74C|74B	GA19582	GA14679	10,614,587–10,623,707
pSCCH	70D|70C	GA20939	GA12507	13,935,508–13,937,677
dSTAR	70B|76C	GA17716	GA17559	14,808,285–14,829,224
dSCCH	78A|78B	GA10355	GA13882	15,622,020–15,654,501
dSCTL	79B	GA13539	GA24132	17,144,949–17,149,139
dHYSC	79B|79C	GA21720	GA24112	17,541,273–17,545,039
dHYST	79D|80A	GA30270	GA10842	17,722,064–17,725,256

a *p:* proximal and *d:* distal. The order of arrangements is from ancestral to derived.

Chromosomal inversions are known to suppress recombination between arrangement heterokaryotopes (Sturtevant and Beadle 1936; Andolfatto *et al.* 2001). Recombination is a fundamental evolutionary process having a strong impact on the genome-wide pattern of nucleotide variation and allelic combinations. Reduced recombination may inhibit the accumulation of deleterious recessive mutations (Nei *et al.* 1967; Ohta and Kojima 1968), trap combinations of positively interacting alleles (Dobzhansky 1950; Wallace 1968; Charlesworth and Charlesworth 1973) or capture a set of genes conferring greater fitness to a particular local habitat while preventing exchange with migrant alleles (Kirkpatrick and Barton 2006; Ayala *et al.* 2013). Suppressed recombination between chromosome arrangements has been documented extensively (Dobzhansky and Epling 1948; Roberts 1976; Ishii and Charlesworth 1977), yet evidence suggests that this is nonuniform across an inversion, as genetic flux among arrangements has been observed within the boundaries of inversion breakpoints resulting from gene conversion or of double crossovers (Kovacevic and Schaeffer 2000; Andolfatto *et al.* 2001; Schaeffer *et al.* 2003; Kennington and Hoffmann 2013). Thus, the effect of inversion polymorphisms on the fine-scale rate of recombination at a chromosome-wide level is not well understood.

The polymorphic third chromosome arrangement system of *D. pseudoobscura* presents a unique opportunity to investigate predictions from recent theory of stabilizing selection on codon usage because of the altered recombinatorial landscape mediated by inversions. Here, we produce fine-scale estimates of the recombination rate between gene arrangements, examine the pattern of synonymous and nonsynonymous polymorphism, and test for the presence of selection acting on codon usage by using full third chromosome sequences of 47 *D. pseudoobscura* individuals representative of five different gene arrangement backgrounds. Previous studies mapping recombination rates between chromosome inversions in *Drosophila* have involved complex crossing schemes of inversion heterokaryotypes or relied on estimating frequencies of double recombination events among progeny with genotypic markers separated by several hundred kilobases (Mestres *et al.* 1998; Stevison *et al.* 2011). In this study, we simply analyze a dense set of polymorphic sites across the chromosome, allowing us to construct fine-scale recombination maps inferred from the pattern of linkage between pairs of single-nucleotide polymorphisms (SNPs). By taking a high-throughput sequencing approach, we are able to identify more than 1 million SNPs among the 47 individuals across the third chromosome, permitting us to consider a large site frequency spectrum (SFS). This rich data set allows us to test whether selection acts on codon usage on the third chromosome and directly investigate how variation in recombination rates affects the intensity of selection.

We find that recombination is significantly suppressed within the inverted regions and the level of synonymous site diversity is elevated between gene arrangements in the overlapping inverted regions. In contrast, both the rate of recombination and the level of nucleotide diversity at synonymous sites are consistent between inversion breakpoints within individual gene arrangements. We find that rates of synonymous and nonsynonymous polymorphism differ between amino acids. The pattern of codon usage bias and strength of selection acting on synonymous sites appears similar between gene arrangements despite varying levels of recombination, effective population sizes, demographic histories, and the relative frequencies of the gene arrangements in natural populations. In contrast to several studies in *D. melanogaster*, but in agreement with recent work, we do not find a significant reduction in either the magnitude of codon bias or the efficacy of natural selection acting on codon usage in regions of suppressed recombination in *D. pseudoobscura*. We conclude that codon usage is likely under stabilizing selection and provide empirical evidence that supports recent theoretical models (Charlesworth 2013).

## Materials and Methods

### Fly stocks

The genomes of 43 *D. pseudoobscura* strains were sequenced for this study. These strains were derived from individuals collected from Mount St. Helena, CA (six strains collected by Wyatt W. Anderson, University of Georgia), James Reserve, CA (12 strains collected by Wyatt W. Anderson, University of Georgia), Santa Cruz Island, CA (one strain collected by Luciano Matzkin, University of Alabama, Huntsville, AL), Kaibab National Forest, AZ (eight strains collect by S.W.S.), Davis Mountains, TX (11 strains collected by S.W.S.), and San Pablo Etla, Mexico (five strains collected by Therese A. Markow, University of California San Diego). These 43 strains were made isochromosomal for the third chromosome using either the Blade or Lobe balancer strains (Dobzhansky and Queal 1938). The genome sequences on Muller Elements A, A/D, B, and E are expected to be a mixture of wild and balancer strain chromosomes and were not considered further in this study. The genome sequences of an additional four *D. pseudoobscura* strains also were included in this study and were collected from Mather, CA (one inbred strain from the Drosophila Species Stock Center, next-generation sequence 75 bp reads, Mohamed Noor, Duke University), Mesa Verde, CO (one inbred strain, Reference Genome MV2−25, collected by Wyatt W. Anderson, whole-genome shotgun sequence; Richards *et al.* 2005), and Bosque del Apache, NM (two strains collected by Bryant McAllister, University of Iowa, next generation sequence 75 bp reads, Mohamed Noor, Duke University). For an outgroup, the genome of one *D. miranda* strain also was included (strain SP138 collected from Spray, OR kindly provided by Mohamed Noor, Duke University, Illumina 75 bp reads; McGaugh *et al.* 2012). The *D. pseudoobscura* strains carry one of five widespread third chromosome gene arrangements: 15 AR, 8 ST, 9 PP, 8 TL, and 7 CH arrangements.

### Illumina library construction and sequencing

Genomic DNA samples from a single male fly of each strain were purified using QIAGEN DNAeasy Blood and Tissue Kit following the manufacturer recommended instructions. The DNA isolation included an RNAse digestion step.

High molecular weight double-strand genomic DNA samples were constructed into Illumina paired end libraries according to the manufacturer’s protocol (Illumina Inc.) with modifications as described here. In brief, 100−400 ng of genomic DNA in 50 µL of volume of low TE buffer was sheared into fragments of approximately 300 base pairs with the Covaris S2 or E210 system (Covaris, Inc. Woburn, MA). The setting was 10% Duty cycle, Intensity of 4200 Cycles per Burst, for 80 sec.

Fragments were processed through DNA End-Repair in 100 μL containing sheared DNA, 10 μL of 10X buffer, 5 μL of EndRepair Enzyme Mix, and H_2_O (NEB*Ne*xt End-Repair Module; Cat. No. E6050L) at room temperature for 30 min; A-tailing was performed in 50 μL containing End-Repaired DNA, 5 μL of 10X buffer, 3 μL of Klenow Fragment (NEB*Ne*xt dA-Tailing Module; cat. no. E6053L) at 37° for 30 min, with each step followed by purification using QIAquick PCR purification kit (cat. no. 28106). Resulting fragments were ligated to Illumina multiplexing PE adaptors using the NEB*Ne*xt Quick Ligation Module (cat. no. E6056L). Ligation was incubated at room temperature for 30 min in 50 μL of reaction containing 10 μL of NEB*Ne*xt Quick Ligase Module (5X buffer), 2 μL of ligation of the Illumina multiplexing PE adaptors (15 µM), and 3 μL of NEB*Ne*xt Quick Ligase. After adapter ligation, the library was purified with QIAquick PCR purification kit (cat. no. 28106) and eluted into 20 μL of H_2_O. The library was then amplified in 50 μL of polymerase chain reaction (PCR) containing 20 μL of library DNA, 1 μL of Illumina PE 1.0, and 1 μL of Illumina IBC1-12 index primers (15 µM), and 25 μL of 2x Custom Platinum PCR Supermix HiFi (Life Technologies, cat. no. A12125) and 3 μL of H_2_O. The standard thermocycling for PCR was 30 sec at 95° for the initial denaturation followed by 8 cycles of 15 sec at 95°, 15 sec at 60°, and 30 sec at 70° and a final extension of 5 min at 70°. A 1.8× volume of Agencourt XP Beads (Beckman Coulter Genomics, Inc.; cat. no. A63882) was used to purify the PCR products. Library size distribution and quantification were analyzed using the Agilent Bioanalyzer 2100 DNA Chip 7500 (Agilent Technologies; cat. no. 5067−1506). Libraries were diluted to 10 nM using 10 mM Tris buffer with 0.1% Tween 20. Five libraries were pooled equally and passed for Illumina sequencing. Shotgun DNA libraries were sequenced on Illumina’s HiSequation 2000 system according to the manufacturer’s specifications. In brief, sequencing libraries were quantified with an Agilent 2100 Bioanalyzer. Cluster generations were performed on an Illumina cluster station. A total of 101 cycles of sequencing were carried out with five barcoded libraries per flow cell lane. Sequencing analysis was first done with the Illumina analysis pipeline. Sequencing image files were processed to generate base calls and phred-like base quality scores and to remove low-quality reads. The reads for the 43 strains have been deposited in the short read archive at the National Center for Biotechnology Information (accession numbers SRX204748-SRX204792, excluding SRX204786 and SRX204788).

### Analysis of high-throughput sequencing reads and final dataset

The 101-bp next-generation sequencing reads were aligned to the *D. pseudoobscura* reference strain FlyBase version 2.27 using BWA (Li and Durbin 2009) with default parameters. GATK (McKenna *et al.* 2010) software was used to remove duplicate sequence reads, recalibrate base quality scores, and locally realign regions around indels for BWA alignments (Depristo *et al.* 2011).The reference strain carries the AR arrangement (Richards *et al.* 2005). SNPs relative to the reference strain were called with GATK (McKenna *et al.* 2010). SNPs with a quality score less than 30 were filtered out of the data set. Nucleotide sites with coverage less than two were filtered from the aligned read data set. On average, 96.066% of nucleotide sites on the third chromosome met our filtering criteria for each individual *D. pseudoobscura* strain (see Supporting Information, Table S1). Because our crossing scheme produced individuals isogenic for the third chromosome, we discarded sites called as heterozygous as they are likely misaligned reads located in highly repetitive regions. Of all polymorphic sites, 7.7–10.9% were called as heterozygous and we found no evidence for clusters of such sites that might suggest particular regions resisted becoming isogenic (Langley *et al.* 2012; see Table S2).The set of SNPs were used to construct an assembled sequence that incorporated nucleotide differences from the reference sequence.

Using the *D. pseudoobscura* reference annotation version 2.27 available from FlyBase, we extracted the coding sequences of each gene. We used SAMtools to generate pileup files from the BAM files (Li *et al.* 2009). The pileup files were used to determine the coverage distributions for each of the strains. Genes that had coverage levels above the 99% confidence interval (see Table S3) were excluded from codon bias analysis because this could indicate the presence of undescribed paralogous genes or the misalignment of nearly identical reads (see Table S4 for list of genes). After filtering, our final dataset included 2798 genes.

### Construction of fine-scale recombination maps

Because recombination only occurs in females, the population scaled recombination rate is defined as *ρ* = *2N_e_r*. We used the program package LDhelmet (Chan *et al.* 2012) to compute fine-scale estimates of *ρ* for all 47 third chromosomes together, as well as independently for each arrangement. LDhelmet follows the same reverse-jump Markov Chain-Monte Carlo approach of other statistical methods, such as LDHat (McVean *et al.* 2004), with several key modifications that improve the accuracy and robustness of recombination rate estimates. Missing data can be handled at no additional computational cost, because the two-locus likelihoods are computed by solving a system of recursion relations. Thus, missing data were not filtered from the input. For each dataset, we generated haplotype configurations with the recommended window size of 50 SNPs (Chan *et al.* 2012). Likelihood lookup tables were computed for the total dataset and each arrangement separately, using independent estimates of the mutation rate parameter, *θ*, obtained from all noncoding sites as a prior (*θ*_Total_ = 0.0134, *θ*_AR_ = 0.0064, *θ*_ST_ = 0.0051, *θ*_PP_ = 0.0056, *θ*_TL_ = 0.0071, *θ*_CH_ = 0.0065). The sampling step of LDhelmet also incorporates a general quadra-allelic mutation model that can handle different rates of mutation for different nucleotides. Inferring ancestral states from *D. miranda*, we followed the procedure of Chan *et al.* (2012) to incorporate the mutational bias observed on the third chromosome. We also computed Pad*é* coefficient tables to improve the accuracy of the sampling step.

We divided each dataset into blocks of 2000 SNPs that overlapped by 200 SNPs with adjacent blocks. For each block, following the procedure of Chan *et al.* (2012), we ran LDhelmet for 3,000,000 iterations after a burn-in of 300,000 iterations. As suggested for *D. melanogaster*, we used a block penalty of 50 (Chan *et al.* 2012). After the reverse-jump Markov Chain-Monte Carlo was completed, the map of the third chromosome for each arrangement was constructed by concatenating adjacent blocks after removing 200 SNPs from the end.

### Estimates of codon bias, diversity, and polymorphism

The effective number of codons (*ENC*) codon adaptation index (*CAI*), and frequency of preferred codons (*Fop*) were estimated for each gene in each arrangement using the software package CodonW (Peden 1999). Custom Python scripts were used to estimate the numbers of nonsynonymous and synonymous sites, diversity (*π*_S_) and polymorphism for each gene within each arrangement (Nei 1987). We also counted the number of synonymous and nonsynonymous segregating sites independently for each amino acid to compute the Watterson (1975) estimator of *θ* using a custom Python script. Finally, a custom Python script was used to estimate Tajima’s *D* statistic (Tajima 1989) using every site on the third chromosome for each arrangement.

### Measuring the strength of association between conserved sites and major codons

The strength of association between conserved sites and major codons for an amino acid can be computed as an odds ratio, denoted here as ψ (Drummond and Wilke 2008; Porceddu *et al.* 2013). As in Akashi (1994), 2 × 2 contingency tables are first constructed for each amino acid from the counts of major and minor codons observed at evolutionary conserved or variable sites. For an amino acid type *i*, ψ is computed as

$$\hat{\psi }=\frac{ad}{bc},$$

where *a* is the number of major codons that encode *i* at a conserved site, *b* is the number of major codons that encode *i* at a variable site, *c* is the number of minor codons that encode *i* at a conserved site and *d* is the number of minor codons that encode *i* at a variable site. In a gene, counts for all amino acids can be combined using a Mantel-Haesenzl procedure (Akashi 1994). ψ was computed for all genes using a custom Python script.

### Estimating the strength of selection at synonymous sites

The method of Zeng and Charlesworth (2009) was used to estimate the strength of selection on codon bias, as well as mutational and demographic parameters. All analyses were conducted with a program kindly provided by Kai Zeng. The parameters are estimated from the SFS, and the model can account for departures from population equilibrium and the demographic effects of a recent population expansion. The full model, denoted as L_1_, includes the following parameters: *θ*_01_, *θ*_10_, *γ*, *g*, *τ*_i_. At equilibrium *θ*_01_ = 4*N_e_µ*_01_, where *µ*_01 i_is the mutation rate between allele *a_0_* and allele *a_1_*, and *θ*_10_ = 4*N_e_µ*_10_, where *µ*_10 i_is the mutation rate between allele *a_1_* and allele *a_0_*_._ The bias between these two mutation rates is represented by the parameter *κ*. *γ* = 4*N_e_s*, where *s* is the selection coefficient. When population expansion is included, *g* = *N_e_*_i_/*N_e_*, where *N_e_*_i_ represents the population size after the *i*-th change in population size and *N_e_* is the effective population size prior to any change. Furthermore, *τ*_i_ = T/(2*N_e_*_i_), where T is the number of generations after the *i*-th change in population size. In the case of a one-step population increase, *i* = 1. In the case of constant population size, *g* = 1 and *τ*_i_ approaches ∞, and thus the model reduces to a simpler form, denoted as L_0._ Additionally, one can reduce the model by fixing parameters such as setting *γ* = 0, in the case of no selection. The log-likelihood of the data under any of the models can then be compared using the χ^2^ test, as in Zeng and Charlesworth (2009), Haddrill *et al.* (2011), and Campos *et al.* (2013). The parameter space was searched using the simplex algorithm of Press *et al.* (1992). To initialize the algorithms, multiple random start points were used and were iterated until they converged. The ML estimates are the result of 10000 independent runs of the program.

## Results

### Genomic sequence data

The mean coverage of the Illumina HiSeq2000 reads for the 47 *D. pseudoobscura* strains on the third chromosome varied from a low of 31× to a high of 57×. Sites on the third chromosome homozygous for the alternative base relative to the reference sequence (AR strain MV2-25) occur at frequency of 0.0066 in AR, 0.0093 in ST, 0.0138 in PP, 0.0136 in CH, and 0.0147 in TL. Across the third chromosome, we identified 1,116,491 SNPs. The numbers of SNPs identified within each arrangement are as follows: 508,720 (AR), 327,079 (ST), 391,194 (PP), 464,651 (TL), and 396,485 (CH). An overestimation of rare variants may be produced by call-based genotype inference; however, the mean coverage in our data set is greater than the greatest mean coverage that produced negligible bias in a recent simulation study (Han *et al.* 2013). Consistent with previous studies analyzing the SFS in *D. pseudoobscura* (Schaeffer *et al.* 2003; Machado *et al.* 2007; Haddrill *et al.* 2011), we observe a slightly negative Tajima’s *D* across the chromosome (Table S5), indicating a skew toward low-frequency variants.

### Recombination between chromosome arrangements is suppressed

**Figure 2:**
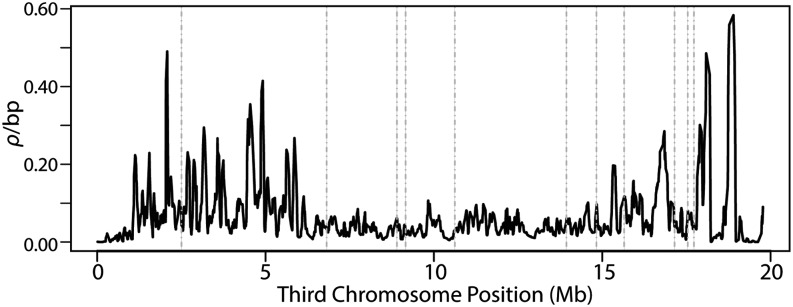
The population scaled recombination rate (*ρ*) of the third chromosome estimated across five gene arrangements of *D. pseudoobscura*. The line is fitted using a cubic smoothing spline with a smoothing parameter of 0.005 and is the mean estimate of *ρ* after 3,000,000 iterations of LDhelmet. The order of sites is according to the Arrowhead reference strain and vertical dashed lines represent the location of inversion breakpoints.

**Figure 3:**
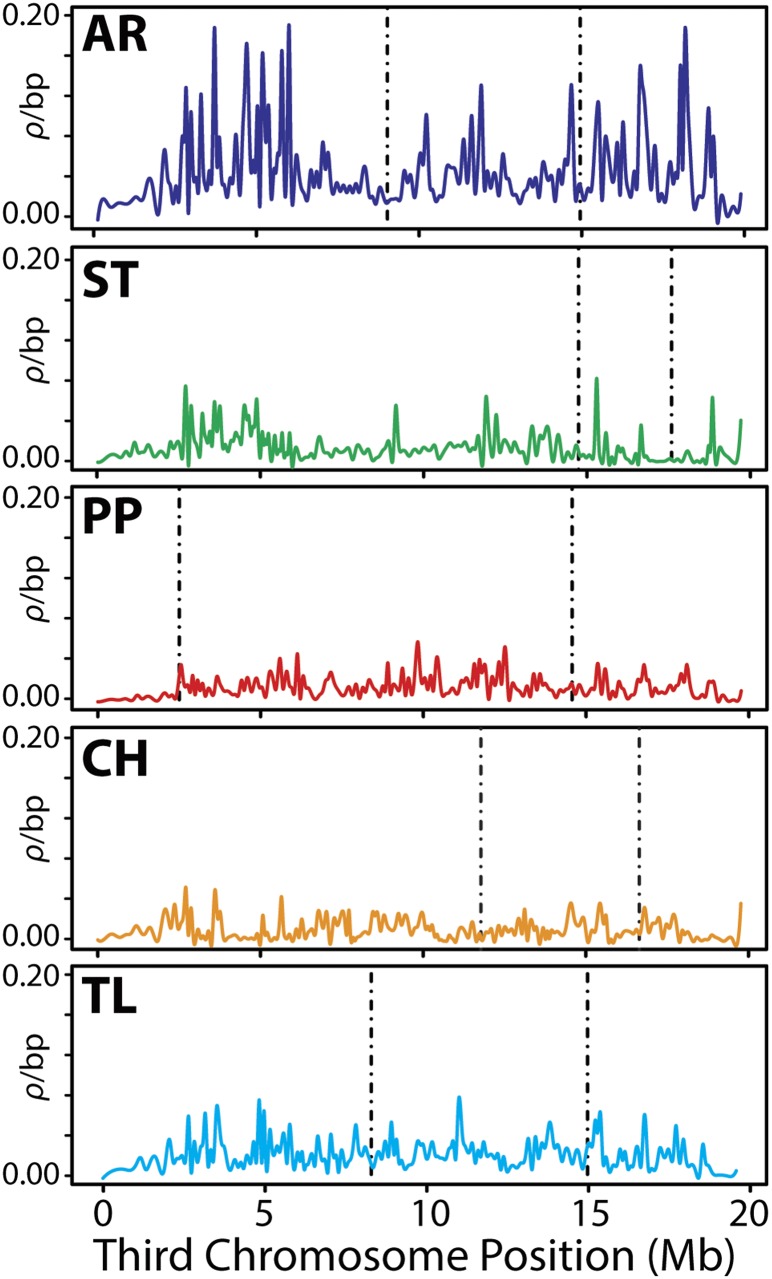
The population scaled recombination rate (*ρ*) for each individual third chromosome arrangement. The line is fitted using a cubic smoothing spline and is the mean estimate of *ρ* after 3,000,000 iterations of LDhelmet. The order of sites is according to the gene order of each arrangement. Vertical dashed lines represent the location of breakpoints of the inversion leading to the arrangement. The scale of the y-axis is the same for all arrangements. AR, Arrowhead; ST, Standard; PP, Pikes Peak; CH, Chiriachua; and TL, Tree Line.

From the sets of inferred SNPs, we used the software package LDhelmet (Chan *et al.* 2012) to construct fine-scale maps of the population scaled recombination rate, *ρ* = *2N_e_r*, for all individuals (Figure 2) and separately for each of the five gene arrangements (Figure 3). *r* is the recombination rate between base pairs per generation and *N_e_* is the effective population size. Because recombination does not occur in *Drosophila* males, both *r* and *N_e_* are multiplied by 2 instead of 4.

Between all gene arrangements, *ρ* across the entire chromosome is estimated to be 0.052/bp (Table 2), which is comparable with whole-chromosome estimates of *ρ* reported in an African *D. melanogaster* population (Rwanda), but greater than estimates in a North American *D. melanogaster* population (Raleigh) obtained using the same method (Chan *et al.* 2012). Considerable heterogeneity in the recombination rate is observed across the chromosome, with *ρ* values up to fivefold greater than the chromosomal average detected in several localized hotspots and greater than 100-fold reductions in *ρ* observed in other regions. Between inversion breakpoints, the average rate of recombination is significantly suppressed compared to the chromosome average (*P* < 0.05, one-sided Wilcoxon rank-sum test, Table 2).On the other hand, the average recombination rate is elevated in the centromeric and telomeric chromosome regions flanking the inversion breakpoints, with *ρ* values of 0.058/bp and 0.056/bp respectively.

**Table 2:** Estimates of the average population scaled recombination rate (*ρ*) for different regions of third chromosome arrangements

Arrangement	*ρ*_Chromosome_	*ρ*_Inverted_	*ρ*_Non-Inverted_	*W**^a^*	*P**^b^*
Total	0.0515	0.0454	0.0537	958310	0.04742*^c^*
AR	0.0607	0.0607	0.0607	936044	~1
ST	0.0214	0.0267	0.0193	1100026	~1
PP	0.0237	0.0298	0.0146	1535837	~1
TL	0.0384	0.0466	0.0343	1574176	~1
CH	0.0183	0.0201	0.0175	956029	~1

AR, Arrowhead; ST, Standard; PP, Pikes Peak; TL, Tree Line; CH, Chiriachua.

a *W* Test statistic from a one-sided Wilcoxon rank-sum test.

b *P* value for a one-sided Wilcoxon rank-sum test with *H_a_*: ρ_Inverted_<ρ_Non-Inverted_).

c Indicates a significant lower rate in inverted regions compared with noninverted regions.

In the fine-scale recombination mapping performed separately for each gene arrangement (Figure 3), no significant reduction in the rate of recombination is detected between inversion breakpoints. In fact, the average value of *ρ* is greater within inverted regions compared with regions located outside the breakpoints (Table 2). The greatest chromosome-wide average recombination rate is detected in the AR arrangement (*ρ_Avg_=*0.061/bp), whereas the lowest is observed in the CH arrangement (*ρ_Avg_* = 0.018/bp). The greatest increase of *ρ_Avg_* from noninverted regions to regions within inversion breakpoints is observed within the PP arrangement, 0.015/bp to 0.030/bp.

### Identification of major and minor codons

Major codons were identified by estimating the relative frequency of each synonymous codon encoding a particular amino acid across our set of third chromosome genes. Preferential use of a major codon was observed for all amino acids (Table 3). The relative frequencies of major and minor codons are similar to that observed by Akashi and Schaeffer (1997), where only 22 genes in *D. pseudoobscura* were analyzed (*P≈*1*,r=*.992). A strong GC bias is observed in the third nucleotide position. All C-ending codons, and all but three G-ending codons are identified as major, a finding identical to a recent study using the second chromosome of *D. pseudoobscura* (Kliman 2014).

**Table 3:** Major codons identified on the third chromosome of *D*. *pseudoobscura*

Amino Acid	Codon	%	*G**^a^*	Amino Acid	Codon	(%)	*G**^a^*
Ala	GCT	16.48		Leu	TTA	2.96	
	GCC	**49.48**	27.84		TTG	14.76	
	GCA	15.75			CTT	7.58	
	GCG	18.29			CTC	19.38	
Arg	AGA	7.66			CTA	7.24	
	AGG	10.09			CTG	**48.09**	69.91
	CGT	15.10		Lys	AAA	26.52	
	CGC	**37.19**	28.98		AAG	**73.48**	22.94
	CGA	12.71		Phe	TTT	35.10	
	CGG	17.25			TTC	**64.90**	9.02
Asn	AAT	40.99		Pro	CCT	10.87	
	AAC	**59.01**	3.27		CCC	**38.11**	17.04
Asp	GAT	46.25			CCA	22.03	
	GAC	**53.75**	0.56		CCG	28.99	
Cys	TGT	26.29		Ser	AGT	11.47	
	TGC	**73.71**	23.42		AGC	**28.95**	24.22
Gln	CAA	24.57			TCT	7.97	
	CAG	**75.43**	27.12		TCC	22.74	
Glu	GAA	26.93			TCA	7.57	
	GAG	**73.07**	22.12		TCG	21.30	
Gly	GGT	15.62		Thr	ACT	14.27	
	GGC	**49.35**	30.75		ACC	**37.35**	12.03
	GGA	22.75			ACA	20.34	
	GGG	12.23			ACG	28.04	
His	CAT	38.52		Tyr	TAT	35.54	
	CAC	**61.48**	5.32		TAC	**64.46**	8.48
Ile	ATT	31.10		Val	GTT	14.20	
	ATC	**47.99**	11.16		GTC	27.36	
	ATA	20.91			GTA	9.10	
					GTG	**49.34**	37.57

a The *G* test statistic for heterogeneity of synonymous codons within an amino acid group. Values in bold depict the percent usage of the identified major codon within an amino acid group.

### Estimates of codon bias

Several methods exist to estimate the level of codon bias within a gene. These include nondirectional measures such as the “Effective Number of Codons” (*ENC*; Wright 1990) and directional measures such as the “Frequency of Optimal Codons” (*Fop*; Ikemura 1985) and the “Codon Adaptation Index” (*CAI*; Sharp and Li 1987). For each gene and for each arrangement, we estimated the average *ENC*, *Fop*, and *CAI*.

First, we sought to determine whether amino acid composition in genes of the third chromosome contributed to the level of codon usage bias. We adopted a sequence randomization method to randomize the possible synonymous codons at each amino acid site in our set of 2798 genes. After generating 1000 replicates for each gene, we then evaluated *ENC* for both the randomized and observed data. Here, we used *ENC* as a measure of codon bias, because it is nondirectional and the effects of amino acid composition could bias codon usage toward both AT and GC ending codons. We considered amino acid composition as strongly contributing to the level of codon bias in a gene if a significant number (>0.05) of randomized sequences were as or more biased than the observed level. For 153 genes, there was a significant *(P <* 0.05) effect of amino acid composition on the observed level of codon bias and these were removed from subsequent analyses.

**Figure 4:**
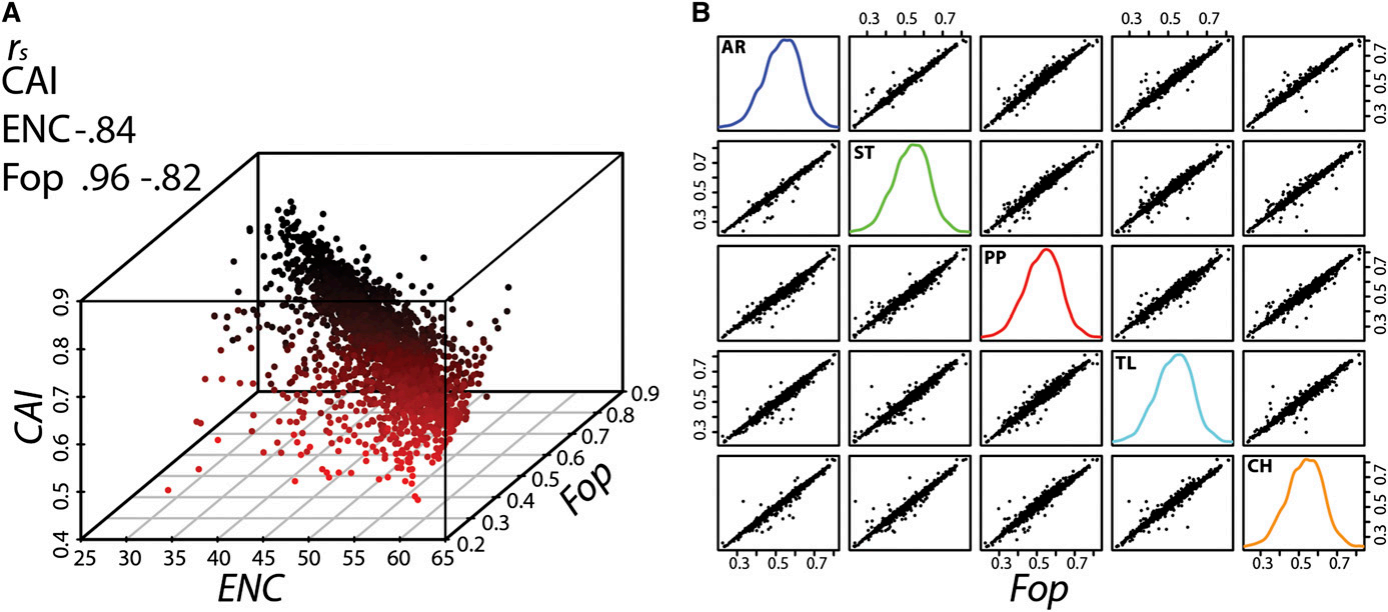
Measures of codon bias on the third chromosome. (A) The correlation and relationship between three measures of codon usage bias (*CAI*, Codon Adaptation Index; *ENC*, Effective Number of Codons; *Fop*, Frequency of Preferred Codon) across all individuals. The matrix in the top left shows Spearman’s correlation coefficient (*r_s_*) between the three measures. (B) The correlation between average *Fop* values for each gene between arrangements. Off-diagonal plots show pairwise comparisons and plots on the diagonal depict the density distribution of average *Fop* values for genes within each arrangement.

*ENC*, *Fop*, and *CAI* estimates are highly correlated (Figure 4A), and no significant differences are detected in the chromosome-wide mean estimates of all three measures between arrangements (Table 4). Furthermore, a high amount of correlation exists between average *Fop* values for each gene between arrangements (Figure 4B). We also performed pairwise Kolmogorov-Smirnov tests between each arrangement and did not detect any significant differences in the distribution of average *Fop* values for any comparison. The pattern and extent of codon usage bias appears to be similar between the five third chromosome arrangements.

**Table 4:** Estimates of three different measures of codon bias

Arrangement	*ENC* (95% CI)	*Fop* (95% CI)	*CAI* (95% CI)
Total	48.16 (47.93−48.41)	0.531 (0.528−0.534)	0.669 (0.666−0.671)
AR	48.14 (47.89−48.38)	0.532 (0.529−0.535)	0.670 (0.667−0.672)
ST	48.10 (47.86−48.35)	0.532 (0.528−0.535)	0.670 (0.667−0.672)
PP	48.22 (47.98−48.46)	0.529 (0.526−0.533)	0.668 (0.665−0.671)
TL	48.19 (47.95−48.43)	0.530 (0.527−0.533)	0.668 (0.666−0.671)
CH	48.21 (47.97−48.45)	0.531 (0.528−0.534)	0.669 (0.666−0.671)

ENC, Effective Number of Codons; CI, confidence interval; Fop, Frequency of Optimal Codons; CAI, Codon Adaptation Index; AR, Arrowhead; ST, Standard; PP, Pikes Peak; TL, Tree Line; CH, Chiriachua.

### The pattern of synonymous polymorphism on the third chromosome

**Figure 5:**
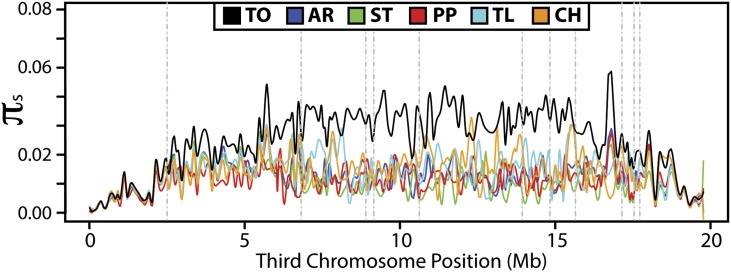
Nucleotide diversity (π_s_, Nei 1987) estimated at synonymous sites for the total dataset and each arrangement. The colored line for each arrangement depicts the LOWESS smoothed line between mean estimates of π_s_ for each gene on the third chromosome. The order of genes is according to the AR reference strain and vertical dashed lines represent the location of inversion breakpoints. AR, Arrowhead; ST, Standard; PP, Pikes Peak; CH, Chiriachua; and TL, Tree Line.

A balanced inversion system is predicted to generate a reduction in the overall level of diversity as a short term effect (Andolfatto *et al.* 2001). As the age of the inversion increases, total nucleotide variability is expected to be similar to that of a panmictic neutral population over much of the inversion due to the effects of mutation, gene conversion, recombination between individuals homozygous for the inversion, and rare double-crossover events between inversion heterokaryotypes (Navarro *et al.* 1997, 2000). Under this model, even with low rates of genetic flux, differentiation is expected to be high near breakpoints, yet arrangements should be nearly indistinguishable toward the center of the inverted region. Inversions have been noted to drastically decrease nucleotide variation near breakpoints (Corbett-Detig and Hartl 2012). In recent studies of *D. melanogaster*, however, the level of polymorphism is found to correlate with the number of inversions present and nucleotide diversity is significantly increased over large stretches of different chromosome arms across inversion breakpoints (Corbett-Detig and Hartl 2012; Pool *et al.* 2012). Furthermore, Wallace *et al.* (2013) observed that both genetic differentiation and nucleotide diversity were significantly increased for a subset of genes within inverted regions of the *D. pseudoobscura* third chromosome. Here, we estimated the nucleotide diversity at synonymous sites (*π*_S_) in each gene for each third chromosome arrangement, as well as for all arrangements together (Figure 5).

Within the inverted chromosome regions, *π*_S_ for all individuals (Total; TO) is significantly greater than *π*_S_ of each of the five individual gene arrangements (Wilcoxon rank-sum test: *W* = 162097, *P* < 2.2 × 10^−16^). For genes located within the inverted regions, the average *π*_S_ value is 0.029 for all individuals, whereas the average *π*_S_ value for each arrangement is as follows: 0.014 (AR), 0.012 (ST), 0.012 (PP), 0.016 (TL), and 0.016 (CH). Wallace *et al.* (2013) similarly reported comparable levels of diversity between different gene arrangements along the medial region of the third *D. pseudoobscura* chromosome but significantly elevated diversity when all arrangements are analyzed together. Outside the inverted chromosome regions, *π*_S_ estimates from the total dataset and for each individual gene arrangement are similar (Figure 5). Between each gene arrangement, the 95% confidence intervals of the mean estimate of *π*_S_ overlap in both the proximal and distal regions outside of the medial inverted regions for each case (Table 5). However, *π*_S_ is significantly different between particular arrangements in the medial region of the chromosome, suggesting that the effective population size differs among gene arrangements within inverted segments. For example, *π*_S_ of ST (0.012) is significantly lower than *π*_S_ of AR (0.014) in the medial inverted overlap region.

**Table 5:** Estimates of recombination (*ρ*) and synonymous site diversity (*π*_S_) for different third chromosome regions

Arrangement	*ρ*	*π*_S_	*ρ*/*π*_S_	*Fop*
TO				
Proximal	0.0577 (0.0547−0.0607)	0.0068 (0.0092−0.0283)	7.232 (5.815−8.649)	0.5259 (0.5202−0.5316)
Inverted	0.0454 (0.0439−0.0466)	0.0289 (0.0282−0.0294)	1.893 (1.668−2.117)	0.5324 (0.5278−0.5371)
Distal	0.0558 (0.0508−0.0609)	0.0119 (0.0107, 0.0133)	6.175 (4.541−7.810)	0.5375 (0.5303− 0.5448)
AR				
Proximal	0.0257 (0.0231−0.0285)	0.0063 (0.0058−0.0069)	6.585 (4.548−8.623)	0.5266 (0.5209−0.5323)
Inverted	0.0670 (0.0652−0.0689)	0.0141 (0.0137−0.0145)	6.965 (6.513−7.416)	0.5339 (0.5293−0.5386)
Distal	0.0488 (0.0405−0.0571)	0.0083 (0.0074−0.0092)	6.863 (5.141−8.584)	0.5386 (0.5313−0.5458)
ST				
Proximal	0.0162 (0.0154−0.0171)	0.0062 (0.0056−0.0068)	3.271 (2.747−3.795)	0.5264 (0.5207−0.5322)
Inverted	0.0233 (0.0225−0.0241)	0.0116 (0.0112−0.0120)	3.178 (2.851−3.506)	0.5336 (0.5288−0.5382)
Distal	0.0083 (0.0069−0.0097)	0.0072 (0.0063−0.0081)	1.456 (1.153−1.761)	0.5386 (0.5312−0.5456)
PP				
Proximal	0.0059 (0.0054−0.0064)	0.0054 (0.0049−0.0059)	1.421 (1.125−1.717)	0.5244 (0.5188−0.5301)
Inverted	0.0278 (0.0271−0.0285)	0.0120 (0.0116−0.0123)	3.661 (3.433−3.884)	0.5308 (0.5262−0.5355)
Distal	0.0121 (0.0103−0.0138)	0.0088 (0.0078−0.0098)	1.547 (1.264−1.829)	0.5369 (0.5297−0.5442)
TL				
Proximal	0.0201 (0.0185−0.0217)	0.0066 (0.0059−0.0072)	4.101 (3.258−4.949)	0.5254 (0.5197−0.5311)
Inverted	0.0437 (0.0427−0.0446)	0.0160 (0.0156−0.0165)	4.005 (3.779−4.231)	0.5309 (0.5262−0.5355)
Distal	0.0148 (0.0122−0.0173)	0.0079 (0.0069−0.0088)	2.059 (1.444−2.673)	0.5359 (0.5287−0.5432)
CH				
Proximal	0.0153 (0.0141−0.0166)	0.0065 (0.0058−0.0071)	2.722 (2.174−3.270)	0.5261 (0.5204−0.5318)
Inverted	0.0199 (0.0192−0.0205)	0.0156 (0.0152−0.0161)	1.901 (1.768−2.034)	0.5319 (0.5272−0.5365)
Distal	0.0066 (0.0052−0.0080)	0.0098 (0.0087−0.0110)	1.130 (0.835−1.424)	0.5373 (0.5301−0.5445)

TO, Total; AR, Arrowhead; ST, Standard; PP, Pikes Peak; TL, Tree Line; CH, Chiriachua.

To gain insight into the relationship between synonymous polymorphism and recombination separately from *N_e_*, we also estimated the mean ratio of *ρ*/*π*_S_ for the proximal, inverted, and distal regions of the chromosome for each arrangement and for all individuals together (Table 5). For the total dataset, this ratio is significantly elevated in the proximal and distal regions outside of the overlapping inversions (*ρ*/*π*_S_ = 7.232 and 6.175 respectively) compared with the inverted region (*ρ*/*π*_S_ = 1.893). On the other hand, this ratio is either increased or not significantly reduced in inverted segments within each third chromosome arrangement.

### Mutational bias and variability in rates of synonymous and nonsynonymous polymorphism

Following Akashi and Schaeffer (1997), we performed a *G*-test for heterogeneity of synonymous codons within each amino acid group (Table 3). Although major codons exist for every amino acid, the relative frequencies of major and minor codons vary widely between amino acids. For instance, the major codon for glutamic acid (GAG) is observed at a frequency of 73.1% (*G* = 22.1), whereas the major codon for aspartic acid (GAC) is only observed at a frequency of 53.8% (*G* = 0.56). We therefore investigated whether the rates of synonymous and nonsynonymous polymorphisms vary between amino acids as well.

**Figure 6:**
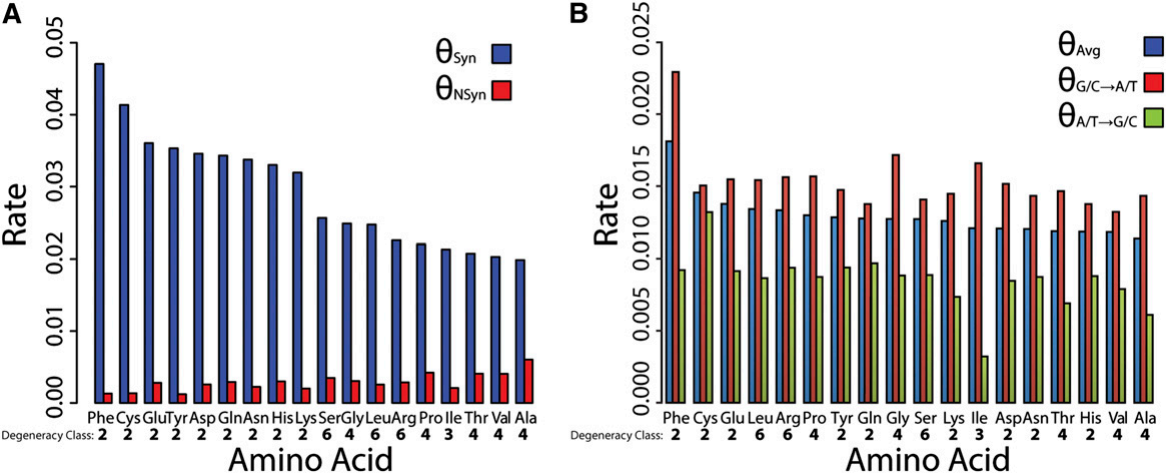
Synonymous and nonsynonymous polymorphism rates for amino acids. (A) The rate of synonymous and nonsynonymous polymorphism (*θ*, Watterson 1975) for each amino acid. The degeneracy class is shown below each amino acid. Stop codons and the onefold degenerate amino acids (Met and Trp) were not considered. (B) The average rate of different transitional mutations for each amino acid. A greater rate of mutation toward AT is observed for all amino acids.

Synonymous and nonsynonymous sites were counted using the approach of Nei and Gojobori (1986), which takes into account the degeneracy of each amino acid. We estimated the rate of synonymous polymorphism per synonymous site and the rate of nonsynonymous polymorphism per nonsynonymous site using the Watterson (1975) estimator of *θ* separately for each amino acid. Nonsynonymous polymorphism rates vary between amino acids, with non-polar alanine estimated to have the greatest (*θ*_NSyn_ = 0.006) and aromatic phenylalanine estimated to have the lowest (*θ*_NSyn_ = 0.001). Interestingly, every twofold degenerate amino acid has an increased rate of synonymous polymorphism compared with the three-, four-, and sixfold degenerate amino acids (Figure 6A). We hypothesized that this effect results from an unequal transition to transversion mutation ratio. If transversions occur less frequently than transitions, the average estimates of *θ*_Syn_ will be lower at sites where synonymous transversions are possible, such as third codon positions of fourfold degenerate amino acids, compared with sites were only synonymous transitions are possible, such as third codon positions of twofold degenerate amino acids. Indeed, if only third codon position transitional mutations are considered, this general trend disappears (Figure 6B), although the twofold degenerate amino acids phenylalanine, cysteine, and glutamic acid still have the greatest three rates of average polymorphism. After inferring ancestral states from *D. miranda*, it is also apparent from analyzing only transitional mutations that there is a greater rate of G/C→A/T mutations than A/T→G/C mutations (Figure 6B). Across all amino acids on the chromosome, the average rate of third codon position G/C→A/T mutations is nearly four times greater than A/T→G/C mutations (*θ*_G/C→A/T_ / *θ*_A/T→G/C_ = 3.949). This bias of mutations toward A/T has been observed in a variety of other organisms, including spontaneous mutation accumulation *D. melanogaster* lines and several divergent taxa of eubacteria (Keightley *et al.* 2009; Hershberg and Petrov 2010; Schrider *et al.* 2013).

Mutational bias, especially in the presence of very weak selection, has been demonstrated to have a major effect on codon usage across the genomes of several species (Palidwor *et al.* 2010; Rao *et al.* 2011). Consistent with several previous studies (Vicario *et al.* 2007; Haddrill *et al.* 2011; Campos *et al.* 2013; Kliman 2014), there appears to be a chromosome-wide mutational bias toward A/T, inferred from the nucleotide composition in noncoding regions (%GC = 43.87) where selective constraints are presumably reduced. This is in contrast to the GC-biased nucleotide composition of coding regions (Table 6) and the observation of all major codons ending in G or C. We estimate that the proportion of third codon positions being a G or C (GC_3_) is 0.654, similar to the estimates of Campos *et al.* (2013) using several autosomal *D. melanogaster* genes.

**Table 6:** Nucleotide composition in coding and noncoding regions

Base	Noncoding, %	Coding, %	Third Codon Position, %
A	28.13	22.19	16.33
T	27.99	22.25	18.29
C	21.96	27.80	32.40
G	21.91	27.76	32.99
% GC	43.87	55.56	65.39

### Codon usage at evolutionary conserved sites

If codon bias results from an effort to increase translational accuracy, the use of major codons is hypothesized to be favored at important and evolutionary conserved amino acid sites (Akashi 1994). For instance, *Escherichia coli* cells starved of asparagine tend to misincorporate lysine when minor asparagine codons are used instead of major codons (Precup and Parker 1987). On the other hand, minor codons may be tolerated at sites in the translated mRNA where errors are less likely to affect the function or folding of the protein product, such as at evolutionary unconserved amino acid positions. We examined the strength of association between major codons and conserved amino acid positions to test for the presence of selection for translational accuracy.

Akashi (1994) devised a statistical test to detect selection on the translational accuracy of a protein coding sequence. The test is built around counting the numbers of major and minor codons at evolutionary conserved and variable sites for each amino acid in a given sequence and determines how likely an association is to have occurred by chance. From these counts, an odds ratio test, denoted as ψ, can be computed that gives the magnitude of the association between major codons and evolutionary conserved amino acid positions. Using *D. miranda* as an outgroup, we defined any site that was monomorphic in all *D. pseudoobscura* individuals and shared with *D. miranda* as conserved. We defined any site that was polymorphic in *D. pseudoobscura* as variable. For each gene, we then computed ψ.

A total of 1887 genes had ψ > 1, meaning there was a greater likelihood of observing an association between a major codon and conserved site than observing an association of a minor codon at a variable site. We find a highly significant linear relationship between ψ and *Fop* (*F* = 5325, *P* < 2.2 × 10^−16^) as well as a strong positive correlation, measured as Spearman’s rank correlation coefficient (0.825). In highly biased genes, major codons are more likely to be used at evolutionary conserved sites, while minor codons are more likely to be used at evolutionary variable sites.

### Estimating the strength of selection at unpreferred and preferred synonymous sites

Several methods have been developed that take advantage of the SFS to estimate the population-scaled selection coefficient, *γ* = *4Ns*, at synonymous sites. (Akashi and Schaeffer 1997; Maside *et al.* 2004; Cutter and Charlesworth 2006; Nielsen *et al.* 2007). Here, we used the method of Zeng and Charlesworth (2009), which allows for demographic changes and does not require the polarization of mutations with an outgroup (see the section *Materials and Methods*). The model assumes the possibility of two alleles, *a_o_* and *a_1_*, at a given nucleotide site and uses the full SFS. If *γ* is positive, then allele *a_0_* is selected for. We constructed allele-frequency spectra with respects to either the counts of major or minor codons to examine the strength of selection acting on preferred and unpreferred mutations. There is increasing evidence that *D. pseudoobscura* has undergone a recent population expansion, which may be more pronounced in the AR arrangement due to recent selection (Schaeffer 2002; Schaeffer *et al.* 2003; Haddrill *et al.* 2011; Wallace *et al.* 2011). The full model of Zeng and Charlesworth (2009), denoted as L_1_, allows for a one-step population expansion whereas a reduced model, L_0_, assumes a constant population size. We first tested the hypothesis of a recent population expansion by comparing the SFS under L_0_ and L_1_ in a maximum likelihood (ML) analysis, following the approach of Campos *et al.* (2013). An L_1_ model with recent population expansion fits the data significantly better than the reduced L_0_ model with constant population size for every third chromosome arrangement using the SFS constructed with regards to major codons (Table 7A). Similar results were obtained using the SFS constructed with regards to minor codons (Table 7B). Hence, we use a model of recent population expansion for subsequent analyses.

**Table 7:** Likelihoods and estimates of mutation and demographic parameters from the site frequency spectrum of preferred (A) and unpreferred (B) mutations

Arrangement	L_0_ ln *L*	NS ln *L*	L_1_ ln *L*	χ^2^: L_1_ *vs.* L_0_	χ^2^: L_1_ *vs.* NS	*γ*_L1_	*κ*_L1_	*g*_L1_	*τ*_L1_
A									
TO	−1647996	−1648361	−1643574	8844	9574	0.788	2.23	3.70	0.026
AR	−1225906	−1224835	−1223811	4190	2048	0.631	1.88	4.28	0.076
ST	−1130330	−1130305	−1130077	506	456	0.433	1.54	3.91	0.042
PP	−1140706	−1140700	−1140549	314	302	0.369	1.46	3.63	1.00
TL	−1170790	−1170911	−1170395	790	1032	0.788	2.25	6.01	0.941
CH	−1153534	−1153802	−1153452	164	700	0.530	1.71	2.37	1.00
B									
TO	−1799586	−1838655	−1796113	3473	42542	−2.42	0.132	24.38	0.003
AR	−1287824	−1298071	−1286418	1406	11653	−3.34	0.161	1.04	1.00
ST	−1168404	−1173753	−1168276	129	5478	−2.03	0.163	1.44	0.925
PP	−1183583	−1189116	−1183569	14	5547	−1.99	0.160	1.19	1.00
TL	−1220625	−1228473	−1220512	113	7961	−2.04	0.147	1.86	1.00
CH	−1198983	−1205659	−1198824	159	6836	−3.08	0.117	0.58	0.997

L_0_, reduced model with no demographic change; NS, reduced model with no selection; L_1_, Full model including selection and population expansion.

If natural selection favors translationally superior codons, the strength of selection should be positive for preferred mutations and negative for unpreferred variants. To test for the presence of positive selection for preferred mutations, we estimated the log-likelihood of the data using a reduced model with *γ* = 0, denoted as NS (*No Selection)*. We then compared the log-likelihood of this null hypothesis to the log-likelihood of the data under the full L_1_ for the SFS constructed with respect to preferred changes. For each arrangement and the total data set, L_1_ fits the data significantly better and *γ* is predicted as >0, indicating the presence of a positive population-scaled selection coefficient for preferred synonymous mutations (Table 7A). Similar results using the SFS constructed with regard to minor codons support γ < 0 for unpreferred changes across all arrangements and the total data set (Table 7B).

For each arrangement, the ML estimate of the mutational bias parameter, *κ*, under the L_1_ model is < 1 for the SFS constructed based on counts of minor codons and >1 for the SFS constructed based on counts of major codons, further supporting the presence of a mutational bias toward unpreferred changes. Furthermore, the absolute values of ML estimates of *γ* are more than triple for unpreferred mutations for each case. These results suggest either a greater intensity of selection chromosome wide against more frequent unpreferred mutations than for preferred changes or a departure from assumptions of the model. ML estimates of the population expansion parameter *g* not only vary widely between the SFS of unpreferred and preferred mutations, but also for arrangements within each SFS (2.37 < *g_Pref_* < 6.01; 0.58 < *g_Unpref_* < 24.38). Additionally, ML estimates of the expansion time parameter τ vary between arrangements (0.026 < *τ_Pref_* < 1.00; 0.003 < *τ_Unpref_* < 1.00). Together, these results indicate different demographic histories of different third chromosome gene arrangements.

**Figure 7:**
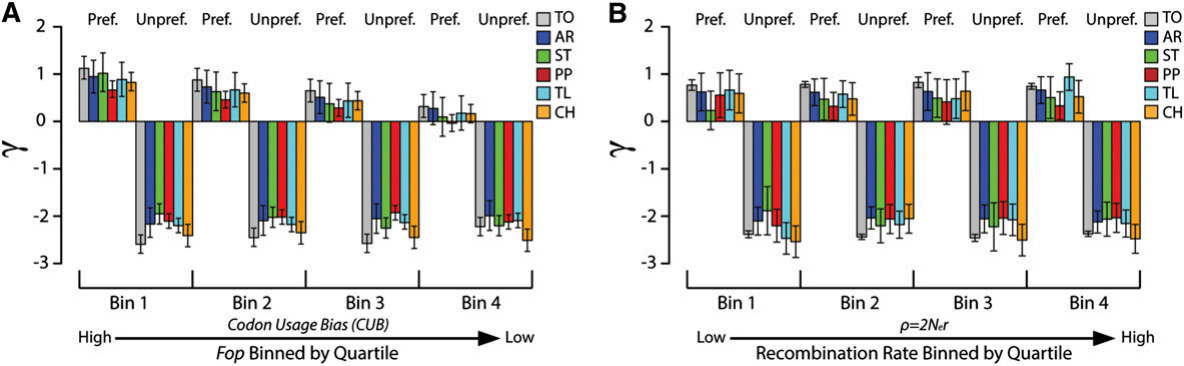
The intensity of selection acting on preferred and unpreferred changes. (A) The population scaled selection coefficient (*γ*) for preferred and unpreferred mutations estimated in genes that vary in levels of codon bias across arrangements. Each bin represents a quartile of the distribution of *Fop* values within each arrangement and is ordered in decreasing levels of codon bias. For example, Bin 1 contains genes with the greatest levels of codon bias and *Fop* values in the top 25% (see Table S6 for the cutoff values for each arrangement). Black bars represent the 95% confidence intervals of the mean ML estimate after 10,000 iterations. (B) The population scaled selection coefficient (*γ*) for preferred and unpreferred mutations estimated in genes with different levels of recombination across arrangements. Each bin represents a quartile of the distribution of average values of *ρ* for genes within each arrangement and is ordered in increasing levels of recombination. For example, Bin 1 contains genes with the lowest level of recombination and average *ρ* values in the lowest 25% (see Table S7 for the cutoff values for each arrangement). Black bars represent the 95% confidence intervals of the mean ML estimate after 10,000 iterations.

It is argued that the optimum level of gene expression, and therefore the need for increased translational accuracy and/or efficiency, may vary widely from gene to gene (Hershberg and Petrov 2008). Hence, the strength of selection acting on preferred and unpreferred sites may vary from gene to gene as well. To test this hypothesis, we estimated *γ* independently for genes in different classes of codon bias. We grouped genes into four different equally sized bins of codon bias by dividing the distribution of *Fop* scores into quartiles for each arrangement (see Table S6 for cutoff values). When all arrangements are analyzed together, there is a significant linear relationship between the class of codon bias and *γ*, with stronger selection acting on more highly biased genes *(F* = 257.2, *P* = 0.00386*)*. Estimates of *γ* between *Fop* bins are not statistically different within individual arrangements as the 95% confidence intervals overlap in every case, but still exhibit the same trend observed in the total data set, where the average *γ* value decreases with the strength of codon bias (Figure 7A). The magnitude of *γ* is greater against unpreferred changes than for preferred changes in all classes of codon bias. Furthermore, the estimates of *γ* for unpreferred changes are not statistically different between classes of codon bias among any of the datasets.

### The relationship between selection, recombination, and codon bias

Using our fine-scale maps, we took the average recombination rate between the beginning and end coordinates of each gene to estimate a mean rate for genes in each independent arrangement and in the total sample. We then grouped genes, independently for each arrangement, into four equally sized bins of recombination rates by dividing the distribution of mean *ρ* values into quartiles (see Table S7 for cutoff values). Again under the full L_1_ model, we then estimated *γ* for the SFS constructed with respects to major or minor codons in genes within each class of recombination for each arrangement as well as for the total sample.

Similar to the results obtained from grouping genes by *Fop*, the magnitude of selection is greater against unpreferred changes than for preferred changes in each bin (Figure 7B). Unlike the previous result, however, *γ* does not significantly change between different classes of recombination. Instead, selection both for preferred changes and against unpreferred changes appears fairly consistent between bins, as the 95% confidence intervals of *γ* overlap across all recombination rate bins for each arrangement. Within individual arrangements, there is not a significant association between the strength of selection either for preferred changes or against unpreferred changes and the rate of recombination. In addition, across the total sample where recombination is genuinely suppressed between arrangements, the strength of selection is not significantly impacted across different rates of recombination.

**Figure 8:**
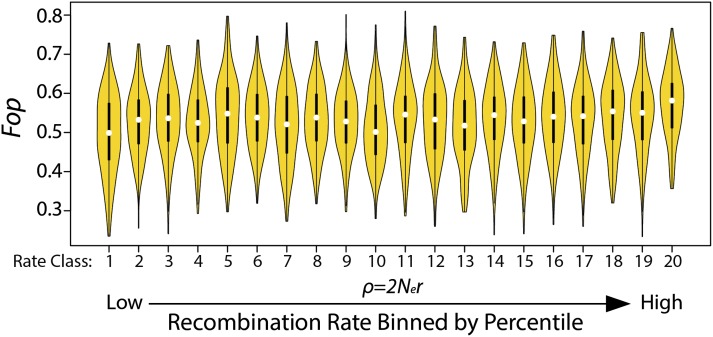
Codon bias (measured as *Fop*) estimated in genes grouped by percentiles of recombination rate (*ρ*) spaced in 5% intervals. The violin plots depict the distribution of *Fop* values within each class of *ρ*. Each class of 5% intervals of *ρ* values is ordered in increasing levels of recombination for the total dataset. For instance, Class 20 contains genes with the greatest level of recombination and average *ρ* values in the greatest 5% (see Table S8 for the cutoff values of each class). The black bars within each violin plot represent the 95% confidence intervals of the mean *Fop* value within each class of *ρ*.

Finally, we analyzed the relationship between the rate of recombination and the magnitude of codon usage bias along the third chromosome. Across all individuals, there is a weak (*R^2^ =* 0.012), yet statistically significant (*F* = 36.61, *P* < 1.63 × 10^−8^) positive linear relationship between *Fop* and the average recombination rate for each gene. Spearman’s rank correlation coefficient (*r_s_*) is 0.097. However, this is likely a result of the small number of genes located in regions of relatively high recombination (*ρ* > 0.2 for 104 genes) and the non-normal distribution of average *ρ* values which violate the assumptions of a simple linear relationship. Instead, if a power transformation (λ = 0.30, determined from a Box-Cox procedure; Supplementary Material) is used on average values of *ρ*, the significance of this relationship disappears *(P <* 0.362). Furthermore, if genes are grouped by percentiles of *ρ* spaced in 5% increments (see Table S8 for cutoff values), no significant differences are detected between mean values of *Fop* (Figure 8), although a slight correlation does exist (*r_s_* = 0.475). In agreement with the recent findings of Kliman (2014), using sites on the second chromosome of *D. pseudoobscura*, we conclude that there is not a significant association between the rate of recombination and either the strength of selection acting on preferred/unpreferred sites or the magnitude of codon bias on the third chromosome.

## Discussion

### Recombination is significantly reduced between third chromosome gene arrangements

Here, as in other recent studies involving *D. pseudoobscura* and *Drosophila* species, we demonstrate that the fine-scale rate of recombination is heterogeneous (Cirulli *et al.* 2007; Kulathinal *et al.* 2009; McGaugh *et al.* 2012). Moreover, we show that the landscape of recombination is drastically modified by chromosomal inversions and that it is significantly suppressed in regions where inversions overlap between heterokaryotypes. Although inversions do not appear to impact local recombination rates within individual gene arrangements, considerable heterogeneity is observed in the average recombination rates when comparing different arrangements.

The chromosome-wide average of *ρ* within AR (*ρ_Avg_* = 0.061/bp) is nearly double that of the arrangement with the next greatest chromosome-wide average (TL, *ρ_Avg_* = 0.038/bp) and over triple compared with the arrangement with the lowest (CH, *ρ_Avg_* = 0.018/bp). This elevated rate of recombination in AR is likely because of the AR arrangement’s demographic history and frequency distribution. Of all the arrangements considered in this study, AR and TL have the largest distribution across the species range as well as the greatest frequency in nature, and thus have the greatest opportunity to form homokaryotypes and freely recombine across the length of the chromosome. AR is nearly fixed in Niche 4 located in the four corners region of the southwestern United States (Figure 1A) where homozygotes would be expected to be found at appreciable frequencies. Furthermore, AR is estimated to be the youngest arrangement, diverging from ST 0.58 million years ago (Mya), but yet despite its age has reached high frequency in a relatively short time (95% confidence interval 0.51−0.65 Mya; Wallace *et al.* 2011). TL is capable of forming homozygotes at appreciable frequencies in Mexican populations, but not as high as AR in the United States.

Although recombination is significantly suppressed in inverted regions of the chromosome where inversions overlap, several hotspots exist outside of the inversion breakpoints near the telomere and centromere that contain peaks of *ρ* that are greater than any observation within inverted segments. Additionally, for the total dataset, the average estimates of *ρ* in noninverted regions are significantly greater than the overlapping inverted regions *(P <* 0.05, Wilcoxon rank-sum test). This finding suggests that genetic exchange and recombination still occurs freely outside of inversions between arrangements. This result is surprising, given that recombination is expected to increase near the telomere yet decrease near the centromere in the presence of an inversion (Navarro *et al.* 1997). Recombination also has been observed to be elevated in regions outside of overlapping inversions in female heterokaryotypes relative to homokaryotypes in several investigations of cross-over rates in *D. melanogaster*, suggesting the interchromosomal effects of inversions may be another possible mechanism increasing recombination outside of breakpoints (Schultz and Redfield 1951; Singleton 1964).

It should be noted that in computer simulations of human sequences, a statistical method similar to LDhelmet, LDhat (McVean *et al.* 2004), has been demonstrated to produce a slight downward bias in the recombination rate estimates when inversions are present (Basatena *et al.* 2013). However, these simulations were performed using just one gene arrangement and detectable differences between the predicted and actual recombination rate only occurred when the inverted chromosome was present at high frequencies (>80%). Furthermore, recombination was modeled according to human genetic variation and inversions were assumed to rise in frequency due to neutral drift, both of which are unlikely scenarios in *D. pseudoobscura*. Because of the large number of third chromosome gene arrangements in *D. pseudoobscura*, inversions not included in this study may potentially impact the estimation of the population-scaled recombination rate. Although very little population structure is observed on the other chromosomes, the overlapping inversions have effectively created a varying number of demes for different genomic regions on the third chromosome. It is unclear how this complex genetic structure affects the estimation of recombination rates from polymorphism data using LDhelmet. Although the relative pattern of suppressed recombination is consistent with the expectation in a polymorphic inversion system, we caution against interpretations of the absolute values obtained from our estimates.

### Nonrandom patterns of synonymous and nonsynonymous polymorphism

Theoretically, each arrangement arose as a single inversion mutation and phylogenies using the SNP data are consistent with this prediction (S. W. Schaeffer, unpublished data). Thus, a sweep-like effect would be expected as the chromosome becomes established in a population, lowering variability within breakpoint regions (Navarro *et al.* 2000). However, we observe consistent levels of synonymous diversity across inversion breakpoints within each arrangement and that diversity is increased across inverted segments when all individuals are analyzed together (Figure 5). These results are in contrast to the neutral theoretical models of Navarro (1997, 2000), which predict low levels of genetic differentiation between arrangements in the central regions of large inversions. In addition, levels of variation are expected to be lower in the youngest arrangements and this is not true in the case of the AR arrangement. Our results also further highlight the importance of considering inversions in population genetics studies, as they can have a dramatic impact on nucleotide diversity estimates across large genomic regions.

Besides variability between regions of high and low recombination, we also observed that average rates of synonymous and nonsynonymous polymorphism differ between amino acids. Our results demonstrate the nonrandom nature of synonymous and nonsynonymous polymorphism and perhaps indicate differential selective constraints for each amino acid. We observe the lowest three rates of nonsynonymous polymorphism in two of the aromatic amino acids (phenylalanine and tyrosine) and cysteine, which is essential for the formation of disulfide bonds and a critical component of protein secondary and tertiary structures. It is hypothesized that purifying selection acts more strongly on such structurally important amino acids and studies in humans have found significant absences of deleterious indel polymorphism compared to genome-wide averages at aromatic amino acid sites (De La Chaux *et al.* 2007). Consistent with this hypothesis, we also observe the greatest rate of nonsynonymous polymorphism in alanine, which is the second smallest amino acid, extremely nonreactive, and rarely involved in protein function (Betts and Russell 2003). These results further demonstrate the nonrandom nature of synonymous and nonsynonymous polymorphism and are consistent with differential selective constraints for each amino acid playing a role in this observation.

A strong bias toward the use of GC nucleotides in codons was observed, with coding regions being GC-rich and all major codons ending with a C or G nucleotide. However, the rate of mutations at synonymous codon positions and nucleotide composition in noncoding regions (Figure 6 and Table 6) indicates a strong bias for G/C→A/T mutations over A/T→ G/C mutations. This mutational bias in favor of AT has been noted numerous times in *D. pseudoobscura* and other *Drosophila* species and occurs in the opposite direction expected from the high GC content of coding regions (Vicario *et al.* 2007). Because this mutational bias is observed across the chromosome in both coding and noncoding regions, it is unlikely that any proposed heterogeneous mutational mechanism, such as positive GC feedback (Fryxell and Zuckerkandl 2000) or misincorporation of G and C bases in early-replicating regions (Eyre-Walker and Hurst 2001) contribute to the observed pattern of codon usage bias in *D. pseudoobscura*.

### Codon usage in regions of suppressed recombination

Although we observe differences in synonymous site diversity between regions of limited recombination, we do not detect a significant association of synonymous codon usage with the recombination rate. Rather, the pattern of biased codon usage is statistically indistinguishable in regions of greatly suppressed recombination from regions of elevated recombination. Contradicting the expectations of Hill-Robertson interference and numerous analyses in *D. melanogaster* (Begun and Aquadro 1992; Kliman and Hey 1993), this result agrees with the recent observations of Kliman (2014), who did not find a significant relationship between recombination and codon bias on the second chromosome of *D. pseudoobscura*.

Significant differences are detected in the mean estimates of *ρ* and *π*_S_ between the medial overlapping inverted regions of the chromosome among gene arrangements. Because both *ρ* and *π*_S_ are scaled by *N_e_*, our results indicate that the relative effective population size differs among gene arrangements as a consequence of suppressed recombination resulting from the presence of overlapping inversions. Contrary to the observations of *ρ* and *π*_S_, we do not observe any significant differences in codon usage between gene arrangements in the medial regions of the chromosome and the mean estimates of *Fop* are similar in every case.

It is possible the recombination rate has recently changed in *D. pseudoobscura* such that the *N_e_* has remained adequate for effective selection or the divergence of the different arrangements is young enough such that the level of biased codon usage has yet to reach equilibrium. When selective pressures change, it can take long evolutionary time periods for codon bias to decay (Sharp *et al.* 2010). However, high levels of linkage disequilibrium are observed between distant markers on the third chromosome (Schaeffer *et al.* 2003), suggesting that selection on codon usage is overcoming the effects of HRI among linked loci and recombination has been suppressed since the inversions arose. Furthermore, the origin of third chromosome arrangements considered in this study varies from young (0.58 Mya, ST→AR) to relatively close to the estimated divergence of *D. pseudoobscura* from *D. miranda* 2 Mya (1.38 Mya, HY→ST; Wallace *et al.* 2011). We do not find a significant difference in codon usage between arrangements, suggesting the age of inversions and HRI as a result of suppressed recombination are unlikely to impact base composition and codon usage on the third chromosome of *D. pseudoobscura*.

### Selection at synonymous sites

Another process hypothesized to increase GC content and affect synonymous codon usage is GC-biased gene conversion (gBGC), where double-strand break repairs are favored toward GC alleles during meiosis in heterozygotes (Marais *et al.* 2001). Although gBGC itself is neutral with respect to fitness, it can mirror the effects of selection by causing GC alleles to have a high probability of fixation (Capra *et al.* 2013). The intensity of gBGC is predicted to be greater in large outcrossing populations and in regions of high recombination (Duret and Galtier 2009). gBGC can be distinguished from natural selection because it affects both coding and noncoding regions, whereas selection acts directly on coding regions and particularly third codon sites (Muyle *et al.* 2011). We observe greater AT content in noncoding regions across the chromosome and the greatest levels of GC in third codon positions. It is therefore implausible that gBGC explains the chromosome-wide patterns of synonymous codon usage in arrangements of *D. pseudoobscura*. Additionally, as discussed earlier we do not observe a significant association between codon usage and recombination that would be expected if biased gene conversion were responsible for base composition and codon bias. The independence of codon usage from the effects of HRI and gBGC has recently been suggested on the X chromosome of *D. melanogaster* (Campos *et al.* 2013). Thus, selection on synonymous sites arises as the likely explanation for the patterns of codon usage in *D. pseudoobscura*.

Using the model of Zeng and Charlesworth (2009), we find that a model including selection fits the SFS of preferred and unpreferred polymorphisms significantly better than a model only including mutational bias, while also taking into account the demographic histories of each arrangement independently. We also find that that the strength of selection acting on synonymous sites does not change as a function of the recombination rate, further supporting our conclusion that selection overcomes the *N_e_* reducing effects of HRI in *D. pseudoobscura*. The intensity of selection does not significantly change between arrangements across classes of codon bias and recombination rates, despite evidence of considerably different effective population sizes, demographic histories and distribution in nature. Selection on codon usage thus appears largely insensitive to the effects of demography, *N_e_*, recombination, biased gene conversion and mutational bias in *D. pseudoobscura*.

Alternative explanations are possible for the apparent lack of association between recombination and codon usage. As suggested by Haddrill *et al.* (2007), even very low frequencies of cross over events may be enough to maintain the efficacy of selection. Although significantly reduced in medial regions of the chromosome, our results show that recombination still takes place between arrangements and double crossover as well as gene conversion events have been observed to occur (Schaeffer *et al.* 2003; Schaeffer and Anderson 2005).

### Stronger selection against unpreferred change than for preferred changes

Because the full SFS is used in the model of Zeng and Charlesworth (2009), *γ* estimated from the SFS constructed with regards to major codons would be expected to equal –*γ* estimated from the SFS constructed with regards to minor codons. Furthermore, *κ* estimated from the SFS constructed with regards to major codons would be expected to equal 1/*κ* estimated from the SFS constructed with regards to minor codons. As expected, we observe a negative selection coefficient and a mutational bias parameter < 1 for estimates using the SFS constructed with regards to minor codons. However, for the entire chromosome and for every arrangement, we find that the magnitude of *γ* is greater for the SFS based on minor codons and unpreferred changes. This may be the result of a true increase in selection against unpreferred changes or a departure from the assumptions of the model. Any algorithm that searches for ML estimates can become trapped in local maximums within the parameter space. However, we used multiple random start points for each parameter and obtained ML estimates after running the algorithm for 10000 independent iterations of each parameter combination.

Significantly stronger selection against unpreferred changes is also found across classes of codon bias and recombination rates (Figure 7), which suggests that there is consistent selection against unpreferred mutations regardless of the level of codon bias in a gene. On the other hand, we find a significant relationship between the strength of selection acting on preferred mutations and increasing levels of codon bias. Selection acts stronger to increase the frequency of major codons for genes with higher levels of codon bias. Furthermore, we observe that the association between major codons and evolutionary conserved sites is strongest in the most highly biased genes. Thus, our data suggests the equilibrium level of optimal codon bias varies between genes.

Hershberg and Petrov (2008) theorized that the function describing the relationship between fitness and codon bias, and therefore the strength of selection in favor of preferred synonymous changes, may differ between genes. For a gene with a low optimal level of codon bias, there may be no fitness benefit to further increase the frequency of major codons in the gene. Hence selection will not act to increase any additional preferred mutations. However, fitness may decrease if unpreferred mutations move the level of codon bias away from the optimum equilibrium value. Here, selection will still act against mutations increasing the number of minor codons in the gene. For a gene with a high optimal level of codon bias, stronger selective pressure will be generated to increase the frequency of major codons and the gene will have to travel through a greater space of suboptimal levels of codon bias until it reaches the optimum. Our results empirically support such a theoretical model of differential selection strength on codon bias. We observe that *γ* is indistinguishable or near-indistinguishable from 0 for preferred mutations in genes with the lowest *Fop* estimates, yet the intensity of *γ* against unpreferred mutations is equally strong across all genes regardless of *Fop*. Genes with the lowest estimates of *Fop* may have low equilibrium optimal levels of codon bias, and therefore additional preferred mutations are effectively neutral.

### Evidence for stabilizing selection acting on synonymous codon usage

Results from this study follow in a long line of literature documenting a preference for G and C ending codons in a background of mutational bias toward A and T across *Drosophila* species (Vicario *et al.* 2007). Despite extensive evidence for selection maintaining high codon bias and GC content in coding regions, the mechanism for selection on this preference in the opposite direction of more frequent AT mutations remains less clear. Furthermore, standard models of selection on codon usage (Li 1987; Bulmer 1991), where the strength of selection scales with *N_e_*, cannot explain the results of this study and the general lack of relationship between codon usage for a species and inferred *N_e_* for a species. Instead, our work provides empirical observations that are consistent with recent models of stabilizing selection causing codon usage bias and support a mechanism for preference of GC-ending codons in an AT-rich mutational background.

Previous work has attributed the apparent lack of association between the effective population size of a species and its level of codon usage bias to synergistic fitness effects at synonymous sites (Akashi 1995; Kondrashov *et al.* 2006). Under this framework, it is predicted that the intensity of selection against unpreferred mutations increases as the proportion of sites with minor codons increases. In contrast to this expectation, we find that the intensity of selection against unpreferred mutations remains constant regardless of the level of codon bias and across chromosome arrangements of the same species that differ in *N_e_*. Additionally, recent simulations demonstrate that the scaled intensity of purifying selection with synergistic fitness effects is in fact proportional to *N_e_* (Charlesworth 2013), which contradicts these previous suggestions and the results presented here. Recently, Charlesworth (2013) developed analytical approximations for parameters of interest in a model of interacting stabilizing selection, mutational bias and drift. A novel conclusion of this model is that the intensity of selection for individual sites under stabilizing selection is effectively independent of *N_e_* if mutational bias is large enough to perturb the population mean of a quantitative trait away from the optimal value by even small amounts.

By definition, selection occurs in the opposite direction of mutational bias under this model. Our results demonstrate that nearly a fourfold mutational bias toward AT exists across the chromosome, which is clearly sufficient to alter the base composition of noncoding regions. As discussed previously, we also find support for the hypothesis that the mean optimal level of codon bias varies among genes and provide evidence that preferred mutations are effectively neutral in genes that may have a low optimal level of codon bias. Numerical results of Charlesworth (2013) demonstrate that a significant pressure of selection is created even when the difference between the mean value of a trait and its optimum is small under a model of stabilizing selection. These analytical and simulation results of a trait evolving under stabilizing selection use biologically plausible parameter values with a level of mutational bias similar to our observations (*κ* = 2−4). For a quantitative trait under stabilizing selection, the intensity of selection is expected to scale with the amount of mutational bias, allowing *γ* to stay constant over a wide range of parameter space. Our data support this prediction, as each chromosome arrangement differs in local recombination rate, demographic history and effective population size, yet the pattern of codon usage and intensity of selection acting on synonymous sites remain consistent between them.

Importantly, the hypothesis of stabilizing selection does not contradict the well-established relationship between gene expression and codon bias. Qian *et al.* (2012) developed evolutionary models from *in vivo* estimates of translation speeds of codons in *Saccharomyces cervisiae* that consider the fitness benefits of translational accuracy and translational efficiency as two separate and sometimes conflicting processes. Genes that are highly expressed have a greater overall need for cellular translational efficiency than genes that are expressed at low levels. Codons that increase the translational accuracy of a gene in some instances decrease the efficiency of translation, and thus different selective pressures for accuracy and efficiency jointly determine the optimal level of codon usage. Here, there may be negative fitness effects to alter the level of codon usage away from the optimal value in either direction. If *D. pseudoobscura* has evolved increased translational efficiency for GC-ending codons, greater GC levels are expected in genes with fitness benefits of increased expression.

For the third chromosome of *D. pseudoobscura*, stabilizing selection can explain the pattern of codon usage and lack of association between selection intensity on synonymous sites and suppressed recombination between arrangements. Stabilizing selection also explains the preference of GC codons in the presence of a high mutational bias toward AT. Because AT mutations are more frequent, this model also explains the consistent and greater intensity of selection acting against unpreferred changes as they have a greater probability of moving the level of codon bias in a gene away from its optimal level. Our results suggest that a role for stabilizing selection acting on codon usage bias should be considered more carefully, especially with the increasing number of population resequencing studies.

## Supplementary Material

Supporting Information
