# Supplementary material for: Evidence for Stabilizing Selection on Codon Usage in Chromosomal Rearrangements of Drosophila pseudoobscura
Source: G3 (Bethesda). 2014 Oct 17;4(12):2433–49. doi: 10.1534/g3.114.014860 (PMC4267939; doi:10.1534/g3.114.014860)
Supplement: Supporting Information [file supp_4_12_2433__index.html]

Evidence for Stabilizing Selection on Codon Usage in Chromosomal Rearrangements of Drosophila pseudoobscura — Supporting Information 

# Evidence for Stabilizing Selection on Codon Usage in Chromosomal Rearrangements of *Drosophila pseudoobscura*

## Supporting Information for Fuller *et al.*, 2014

**Files in this Data Supplement:**

- Supporting Information - Tables S1-S8 and File S1 (PDF, 397 KB)
- Table S1 - Number and fraction of bases with coverage > 2 and quality score > 30 for each strain. (PDF, 139 KB)
- Table S2 - Heterozygous and SNP sites on the third chromosome in 46 genome sequences of *D. pseudoobscura* strains. (PDF, 171 KB)
- Table S3 - 99% confidence intervals of the mean coverage. (PDF, 144 KB)
- Table S4 - Genes with significant high coverage. (PDF, 140 KB)
- Table S5 - Tajima's *D* in *D. pseudoobscura* third chromosome gene arrangements. (PDF, 212 KB)
- Table S6 - Cutoff values of *Fop* bins for each arrangement. (PDF, 176 KB)
- Table S7 - Cutoff values of recombination rate (*ρ*/bp) bins for each arrangement. (PDF, 165 KB)
- Table S8 - Cutoff values for genes grouped by percentiles of *ρ* spaced in 5% intervals. (PDF, 181 KB)
- File S1 - Supplementary Material (PDF, 272 KB)
