# Supplementary material for: Evidence for Stabilizing Selection on Codon Usage in Chromosomal Rearrangements of Drosophila pseudoobscura
Source: G3 (Bethesda). 2014 Oct 17;4(12):2433–49. doi: 10.1534/g3.114.014860 (PMC4267939; doi:10.1534/g3.114.014860)
Supplement: Supporting Information [file supp_g3.114.014860_014860SI.pdf]

G3: Genes | Genomes | Genetics

Evidence for Stabilizing Selection on Codon Usage in Chromosomal Rearrangements of *Drosophila pseudoobscura*

Zachary L. Fuller[*], Gwilym D. Haynes[*], Dianhui Zhu[1] [§] , Matthew Batterton[§], Hsu Chao[§], Shannon Dugan[§], Mehwish Javaid[§], Joy C. Jayaseelan[§], Sandra Lee[§], Mingmei Li[§], Fiona Ongeri[§], Sulan Qi[§], Yi Han[§], Harshavardhan Doddapaneni[§], Stephen Richards[§], Stephen W. Schaeffer[*]

[*] 208 Erwin W. Mueller Laboratory, Department of Biology, The Pennsylvania State University, University Park, PA 16802-5301, Telephone: 814-865-3269

[§] Human Genome Sequencing Center, Baylor College of Medicine, 1 Baylor Plaza, Houston, TX, 77030

([1] current address: Chevron Inc. HOU160/45018A 1600 Smith St. Houston, TX77002)

Corresponding author: Zachary L. Fuller (zlf105@psu.edu)

208 Erwin W.Mueller Laboratory

The Pennsylvania State University

University Park, PA 16801

Telephone: (814) 865-3269 or (814) 867-3344

DOI: 10.1534/g3.114.014860

Table S1   Number and fraction of bases with coverage > 2 and quality score > 30 for each strain.

Strain	Bases with Cov 2 Quality 30	%Bases
AR_DM1005	19111087	96.621
AR_DM1015	19105631	96.593
AR_DM1050	19101359	96.571
AR_DM1056	19083423	96.481
AR_DM1088	19099017	96.560
AR_KB635	19093362	96.531
AR_KB652	19103813	96.584
AR_KB754	19094385	96.536
AR_KB819	19095971	96.544
AR_KB820	19104457	96.587
AR_KB827	19113924	96.635
AR_KB945	19108211	96.606
AR_MSH126	19116131	96.646
AR_MSH51	19110425	96.617
CH_JR198	19015025	96.135
CH_JR20	19021627	96.168
CH_JR272	18999508	96.056
CH_JR356	19006280	96.091
CH_JR377	19009452	96.107
CH_KB888	19012442	96.122
CH_MSH202	19027792	96.199
PP_BdA1134-13	18256001	92.297
PP_BdA1137-10	18353536	92.791
PP_DM1038	19027338	96.197
PP_DM1049	19015640	96.138
PP_DM1054	18997597	96.047
PP_DM1065	19034951	96.236
PP_DM1081	19038581	96.254
PP_DM1084	19046210	96.293
PP_JR83	19007207	96.095
ST_JR138	19086682	96.497
ST_JR158	19088298	96.505
ST_JR209	19092746	96.528
ST_JR72	19109339	96.612
ST_JR84	19089559	96.512
ST_JR91	19085966	96.494
ST_MSH177	19099534	96.562
ST_MSH217	19095487	96.542
TL_MA1959	18118637	91.603
TL_MSH130	18952702	95.820
TL_SCI12-2	19004694	96.083

TL_SPE123_2-3	18979323	95.954
TL_SPE123_5-1	18985208	95.984
TL_SPE123_6-3	19010828	96.114
TL_SPE123_7-1	19013924	96.129
TL_SPE123_8-1	18970717	95.911
Dmir_SP138	16775187	84.811

Table S2 Heterozygous and SNP sites on the third chromosome in 46 genome sequences of *D. pseudoobscura* strains.

Strain	Het 0/1 (%)	Hom 1/1 (%)	Het 1/2 (%)	Total
AR_DM1005	14828 (10.2)	130700 (89.7)	240 (0.2)	145768
AR_DM1015	13541 (9.3)	131142 (90.5)	200 (0.1)	144883
AR_DM1050	13261 (9.1)	131665 (90.7)	179 (0.1)	145105
AR_DM1056	12077 (8.4)	131787 (91.5)	155 (0.1)	144019
AR_DM1088	12508 (8.7)	131840 (91.2)	161 (0.1)	144509
AR_KB635	13799 (9.6)	130496 (90.3)	181 (0.1)	144476
AR_KB652	13702 (9.4)	131878 (90.5)	186 (0.1)	145766
AR_KB754	12714 (8.9)	130679 (91.0)	179 (0.1)	143572
AR_KB819	12613 (8.8)	130130 (91.1)	169 (0.1)	142912
AR_KB820	11859 (8.2)	131991 (91.7)	152 (0.1)	144002
AR_KB827	13344 (9.2)	130830 (90.6)	187 (0.1)	144361
AR_KB945	12472 (8.6)	132022 (91.3)	153 (0.1)	144647
AR_MSH51	13891 (9.6)	131334 (90.3)	205 (0.1)	145430
AR_MSH126	14668 (9.9)	133024 (89.9)	236 (0.2)	147928
CH_JR20	22846 (7.7)	274352 (92.2)	429 (0.1)	297627
CH_JR198	22357 (7.6)	272449 (92.3)	417 (0.1)	295223
CH_JR272	22650 (7.7)	270334 (92.2)	361 (0.1)	293345
CH_JR356	21754 (7.7)	259309 (92.2)	306 (0.1)	281369
CH_JR377	22064 (7.5)	270738 (92.3)	394 (0.1)	293196
CH_KB888	21488 (7.4)	266728 (92.4)	388 (0.1)	288604
CH_MSH202	22843 (7.7)	274812 (92.2)	403 (0.1)	298058
PP_BdA1134-13	29089 (10.5)	246987 (89.3)	415 (0.2)	276491
PP_BdA1137-10	31345 (10.9)	255458 (88.9)	574 (0.2)	287377
PP_DM1038	22436 (7.4)	280484 (92.5)	394 (0.1)	303314
PP_DM1049	22814 (7.5)	280772 (92.4)	431 (0.1)	304017
PP_DM1054	21438 (7.1)	278961 (92.7)	393 (0.1)	300792
PP_DM1065	23950 (7.8)	282607 (92.0)	473 (0.2)	307030
PP_DM1081	24819 (8.0)	283619 (91.8)	535 (0.2)	308973
PP_DM1084	25483 (8.3)	281507 (91.5)	505 (0.2)	307495
PP_JR83	20670 (6.9)	278377 (93.0)	356 (0.1)	299403
ST_JR138	15760 (7.9)	183717 (92.0)	240 (0.1)	199717
ST_JR158	16684 (8.3)	183057 (91.5)	238 (0.1)	199979
ST_JR209	16315 (8.2)	181353 (91.6)	251 (0.1)	197919
ST_JR72	17677 (8.8)	182920 (91)	325 (0.2)	200922

ST_JR84	15488 (7.8)	184040 (92.1)	242 (0.1)	199770
ST_JR91	15869 (8.0)	182346 (91.9)	235 (0.1)	198450
ST_MSH177	17261 (8.6)	184169 (91.3)	233 (0.1)	201663
ST_MSH217	16634 (8.2)	185374 (91.7)	238 (0.1)	202246
TL_MA1959	29569 (10.4)	254859 (89.5)	409 (0.1)	284837
TL_MSH130	25155 (8.0)	289724 (91.9)	390 (0.1)	315269
TL_SCI_12-1	23987 (7.5)	293739 (92.3)	440 (0.1)	318166
TL_SPE123_2-3	23954 (7.5)	296292 (92.4)	398 (0.1)	320644
TL_SPE123_5-1	22076 (6.9)	296615 (93.0)	414 (0.1)	319105
TL_SPE123_6-3	27268 (8.4)	297866 (91.5)	510 (0.2)	325644
TL_SPE123_7-1	27355 (8.4)	297842 (91.4)	535 (0.2)	325732
TL_SPE123_8-1	24242 (7.6)	295852 (92.3)	428 (0.1)	320522

Het 0/1, Site called as heterozygous with the reference base and an alternative base; Hom 1/1, Site called as a SNP difference from the reference base; Het 1/2, Site called as heterozygous with two alternative bases.

Table S3   99% confidence intervals of the mean coverage

Strain	Location	Mean	Median	Mode	Cov_99.5%	Max
AR_DM1005	Davis Mts, TX	59.5	60	61	113	7996
AR_DM1015	Davis Mts, TX	46.4	47	47	94	5299
AR_DM1050	Davis Mts, TX	42.8	43	43	82	3661
AR_DM1056	Davis Mts, TX	31.5	32	32	62	3004
AR_DM1088	Davis Mts, TX	38.6	39	39	78	3801
AR_KB635	Kaibab NF, AZ	40.4	41	41	77	2385
AR_KB652	Kaibab NF, AZ	50.0	51	51	97	4346
AR_KB754	Kaibab NF, AZ	39.5	40	40	77	2662
AR_KB819	Kaibab NF, AZ	34.6	35	35	66	3122
AR_KB820	Kaibab NF, AZ	32.6	33	33	63	2290
AR_KB827	Kaibab NF, AZ	54.7	55	56	107	7955
AR_KB945	Kaibab NF, AZ	40.1	40	41	78	2871
AR_MSH126	Mt. St. Helena, CA	47.3	48	48	92	3948
AR_MSH51	Mt. St. Helena, CA	44.9	45	47	89	7801
PP_BdA1134-13	BosqueDelApache, NM	34.1	36	40	90	1429
PP_BdA1137-10	BosqueDelApache, NM	46.1	49	54	120	2482
PP_DM1038	Davis Mts, TX	39.2	41	42	75	2267
PP_DM1049	Davis Mts, TX	43.7	45	47	84	3425
PP_DM1054	Davis Mts, TX	38.5	40	41	73	3319
PP_DM1065	Davis Mts, TX	50.1	52	54	95	4109
PP_DM1081	Davis Mts, TX	57.1	59	62	101	4200
PP_DM1084	Davis Mts, TX	56.9	59	62	111	3531
PP_JR83	James Reserve, CA	35.0	36	38	68	1780
ST_JR138	James Reserve, CA	35.5	36	36	70	1772
ST_JR158	James Reserve, CA	45.0	45	46	88	3917
ST_JR209	James Reserve, CA	36.1	36	37	75	3425
ST_JR72	James Reserve, CA	55.4	56	57	107	4499
ST_JR84	James Reserve, CA	38.1	39	39	78	1667
ST_JR91	James Reserve, CA	34.1	34	35	64	2659
ST_MSH177	Mt. St. Helena, CA	43.1	44	44	84	2110
ST_MSH217	Mt. St. Helena, CA	44.8	46	46	90	1843
CH_JR198	James Reserve, CA	36.3	37	39	81	1947
CH_JR20	James Reserve, CA	37.3	38	40	75	2355
CH_JR272	James Reserve, CA	42.1	43	45	92	3202
CH_JR4	James Reserve, CA					
CH_JR356	James Reserve, CA	35.3	36	37	78	2205
CH_JR377	James Reserve, CA	34.0	34	35	72	4429

CH_KB888	Kaibab NF, AZ	41.6	43	45	86	3049
CH_MSH202	Mt. St. Helena, CA	38.4	40	41	80	1991
TL_MA1959	Mather, CA	34.0	35	39	94	1418
TL_MSH76	Mt. St. Helena, CA					
TL_MSH130	Mt. St. Helena, CA	39.2	40	41	84	5359
TL_SCI12-2	Santa Cruz Island, CA	47.5	49	51	99	3080
TL_SPE123_2-3	San Pablo Etla, Oaxaca, Mexico	42.8	43	45	97	7232
TL_SPE123_5-1	San Pablo Etla, Oaxaca, Mexico	39.6	41	43	82	2249
TL_SPE123_6-3	San Pablo Etla, Oaxaca, Mexico	51.2	53	56	112	7618
TL_SPE123_7-1	San Pablo Etla, Oaxaca, Mexico	53.5	55	58	128	7555
TL_SPE123_8-1	San Pablo Etla, Oaxaca, Mexico	39.6	41	43	85	3262
Dmir_SP138		14.6	14	17	86	2946

Table S4 Genes with significant high coverage

Gene	FBID_KEY	DROSOPHILA_ORTHOLOGS	LOC_MIN	LOC_MAX	LOC_STR
GA24632	FBgn0246022	-	1597752	1600079	1
GA15282	FBgn0075305	Rs1	2034368	2037130	1
GA24526	FBgn0245917	-	2038979	2039580	-1
GA24524	FBgn0245915	-	2039717	2040495	-1
GA24505	FBgn0245896	-	2576736	2580315	-1
GA24679	FBgn0083525	Or98a	3461540	3464987	1
GA24684	FBgn0246070	-	3505258	3506284	1
GA15652	FBgn0075669	NT5E-2	4828913	4831002	1
GA24437	FBgn0245834	-	4831115	4831843	-1
GA24421	FBgn0245818	-	5535422	5541561	-1
GA21477	FBgn0081464	-	6803237	6803734	-1
GA24775	FBgn0246161	tej	6858394	6860432	1
GA11906	FBgn0071955	CG12917	6878299	6879318	1
GA24778	FBgn0086700	Or46a	6879873	6882468	1
GA14766	FBgn0074793	CG18011	6882805	6886492	1
GA24369	FBgn0245766	-	7508444	7508899	-1
GA24821	FBgn0246206	-	8484241	8485145	1
GA10531	FBgn0070588	CG10737	9895870	9902715	1
GA14679	FBgn0074706	rdgBbeta	10628202	10630184	1
GA24283	FBgn02456	Cpr47Ef	1104570	11048905	-1

	80		6		
GA24280	FBgn0245677	CG14518, CG33725, CG33796	11175169	11175825	-1
GA21260	FBgn0081248	Cyp6a14	13261739	13263324	1
GA24236	FBgn0245635	CG13204	13533162	13536583	-1
GA15589	FBgn0075606	Gr47b	13540481	13541836	-1
GA15365	FBgn0075384	ptc	13682985	13696593	1
GA24978	FBgn0246361	CG7741	14053446	14055483	1
GA24214	FBgn0245613	CG33632, CG33912, CG33764	14218207	14218923	-1
GA12324	FBgn0072370	Gr58c	15426236	15427526	1
GA25040	FBgn0246423	CG30069	16304677	16311492	1
GA21181	FBgn0081169	Ih	17421113	17439461	1
GA24105	FBgn0245504	-	17703813	17707738	-1
GA30269	FBgn0263811	CG42678	17806002	17811813	-1
GA12384	FBgn0072430	CG13590, CG13589	18293905	18294537	1
GA30265	FBgn0263807	Strn-Mlck	18965505	18999327	1

Table S5 Tajima's *D* in *D. pseudoobscura* third chromosome gene arrangements.

**Arrangement**	**Tajima's *D***
Arrowhead (AR)	-.7259
Pikes Peak (PP)	-.2548
Standard (ST)	-.2524
Tree Line (TL)	-.3517
Chiricahua (CH)	-.1317

Table S6   Cutoff values of *Fop* bins for each arrangement

Arrangement	Bin 1 (75-100%)	Bin 2 (50-75%)	Bin 3 (25-50%)	Bin 4 (0-25%)
Total	≥0.5962	0.5359-0.5962	0.4694-0.5359	≤0.4694
AR	≥0.5970	0.5367-0.5970	0.4710-0.5367	≤0.4710
ST	≥0.5976	0.5367-0.5976	0.4708-0.5367	≤0.4708
PP	≥0.5937	0.5342-0.5937	0.4684-0.5342	≤0.4684
TL	≥0.5953	0.5351-0.5953	0.4683-0.5351	≤0.4683
CH	≥0.5964	0.5354-0.5964	0.4703-0.5354	≤0.4703

Table S7 Cutoff values of recombination rate (ρ/bp) bins for each arrangement

Arrangement	Bin 1 (0-25%)	Bin 2 (25-50%)	Bin 3 (50-75%)	Bin 4 (75-100%)
Total	≤0.0232	0.0232-0.0405	0.0405-0.0655	≥0.0655
AR	≤0.0175	0.0175-0.0295	0.0295-0.0486	≥0.0486
ST	≤0.0044	0.0044-0.0100	0.0100-0.0193	≥0.0193
PP	≤0.0070	0.0070-0.0124	0.0124-0.0199	≥0.0199
TL	≤0.0108	0.0108-0.0199	0.0199-0.0296	≥0.0296
CH	≤0.0035	0.0035-0.0082	0.0082-0.0176	≥0.0176

Table S8 Cutoff values for genes grouped by percentiles of ρ spaced in 5% intervals.

Class	Percentile	ρ/bp
1	5%	≤0.0089
2	10%	0.0089-0.0125
3	15%	0.0125-0.0167
4	20%	0.0167-0.0203
5	25%	0.0203-0.0232
6	30%	0.0232-0.0266
7	35%	0.0266-0.0297
8	40%	0.0297-0.0332
9	45%	0.0332-0.0364
10	50%	0.0364-0.0405
11	55%	0.0405-0.0445
12	60%	0.0445-0.0484
13	65%	0.0484-0.0532
14	70%	0.0532-0.0576
15	75%	0.0576-0.0651
16	80%	0.0651-0.0722
17	85%	0.0722-0.0805
18	90%	0.0805-0.0948
19	95%	0.0948-0.1389
20	100%	≥0.1389

File S1

Supplementary Material

Relationship between recombination and codon bias

If a simple linear regression is performed on the average recombination rate (ρ/bp) for each gene and *Fop*, there is a weak yet statistically significant relationship (F=36.61, P=1.63x10^{-8}). The adjusted R-squared value is 0.01258.

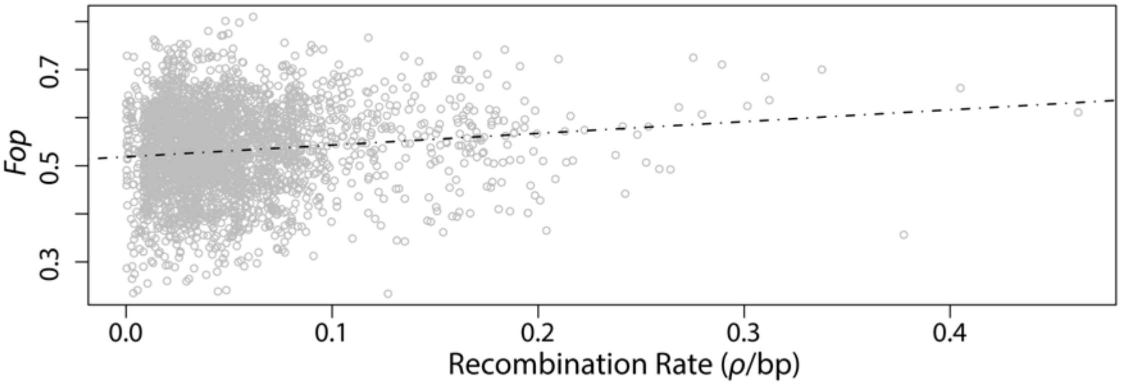

However, there are only 104 genes with average values of ρ greater than 0.2, which is likely influencing the result. Values of ρ do not appear to be normally distributed, which is an assumption of a simple linear regression. To create a more normal distribution of ρ values, a Box-Cox procedure was performed to determine the profile likelihood of the λ parameter for a power-transformation.

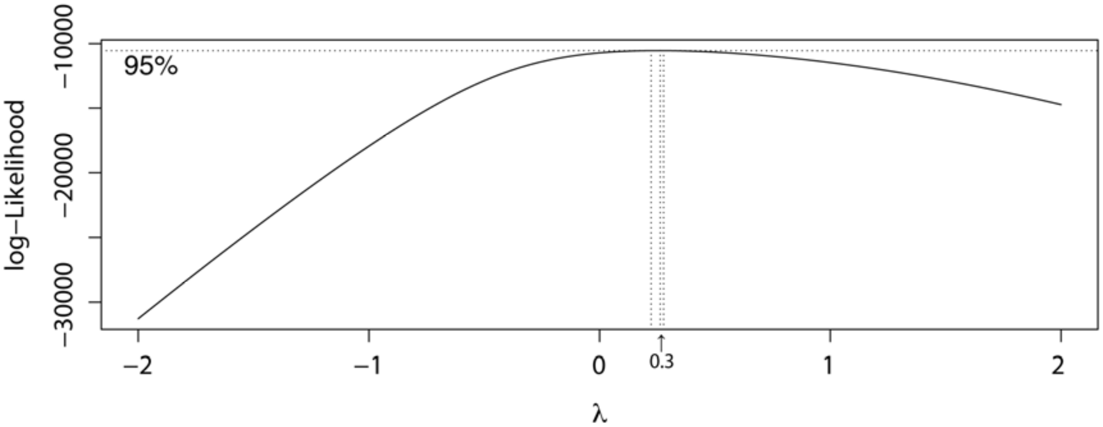

Values of ρ then underwent a power transformation with a λ parameter set to 0.3. For the transformed data, a significant relationship disappears (F=0.83, P=0.3624) and the correlation becomes even weaker (r=0.01249, R^2_{ADJ}=0.01214).

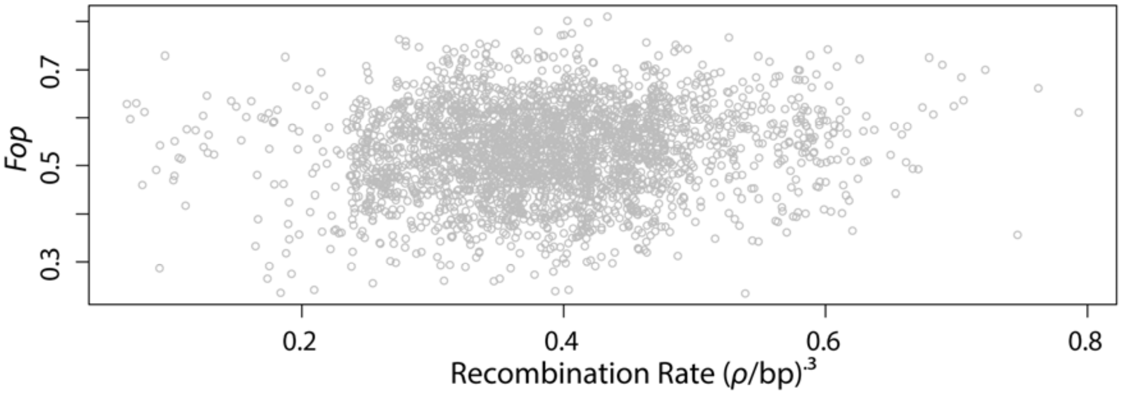

Fop
0.3
0.5
0.7
0.2
0.4
0.6
0.8
Recombination Rate (ρ/bp)^.3
