# Supplementary material for: Evidence for Stabilizing Selection on Codon Usage in Chromosomal Rearrangements of Drosophila pseudoobscura
Source: G3 (Bethesda). 2014 Oct 17;4(12):2433–49. doi: 10.1534/g3.114.014860 (PMC4267939; doi:10.1534/g3.114.014860)
Supplement: Supporting Information [file supp_g3.114.014860_TableS1.pdf]

Table S1 Number and fraction of bases with coverage > 2 and quality score > 30 for each strain.

Strain	Bases with Cov 2 Quality 30	%Bases
AR_DM1005	19111087	96.621
AR_DM1015	19105631	96.593
AR_DM1050	19101359	96.571
AR_DM1056	19083423	96.481
AR_DM1088	19099017	96.560
AR_KB635	19093362	96.531
AR_KB652	19103813	96.584
AR_KB754	19094385	96.536
AR_KB819	19095971	96.544
AR_KB820	19104457	96.587
AR_KB827	19113924	96.635
AR_KB945	19108211	96.606
AR_MSH126	19116131	96.646
AR_MSH51	19110425	96.617
CH_JR198	19015025	96.135
CH_JR20	19021627	96.168
CH_JR272	18999508	96.056
CH_JR356	19006280	96.091
CH_JR377	19009452	96.107
CH_KB888	19012442	96.122
CH_MSH202	19027792	96.199
PP_BdA1134-13	18256001	92.297
PP_BdA1137-10	18353536	92.791
PP_DM1038	19027338	96.197
PP_DM1049	19015640	96.138
PP_DM1054	18997597	96.047
PP_DM1065	19034951	96.236
PP_DM1081	19038581	96.254
PP_DM1084	19046210	96.293
PP_JR83	19007207	96.095
ST_JR138	19086682	96.497
ST_JR158	19088298	96.505
ST_JR209	19092746	96.528
ST_JR72	19109339	96.612
ST_JR84	19089559	96.512
ST_JR91	19085966	96.494
ST_MSH177	19099534	96.562
ST_MSH217	19095487	96.542
TL_MA1959	18118637	91.603
TL_MSH130	18952702	95.820
TL_SCI12-2	19004694	96.083

TL_SPE123_2-3	18979323	95.954
TL_SPE123_5-1	18985208	95.984
TL_SPE123_6-3	19010828	96.114
TL_SPE123_7-1	19013924	96.129
TL_SPE123_8-1	18970717	95.911
Dmir_SP138	16775187	84.811
