# Supplementary material for: Evidence for Stabilizing Selection on Codon Usage in Chromosomal Rearrangements of Drosophila pseudoobscura
Source: G3 (Bethesda). 2014 Oct 17;4(12):2433–49. doi: 10.1534/g3.114.014860 (PMC4267939; doi:10.1534/g3.114.014860)
Supplement: Supporting Information [file supp_g3.114.014860_TableS2.pdf]

Table S2 Heterozygous and SNP sites on the third chromosome in 46 genome sequences of *D. pseudoobscura* strains.

Strain	Het 0/1 (%)	Hom 1/1 (%)	Het 1/2 (%)	Total
AR_DM1005	14828 (10.2)	130700 (89.7)	240 (0.2)	145768
AR_DM1015	13541 (9.3)	131142 (90.5)	200 (0.1)	144883
AR_DM1050	13261 (9.1)	131665 (90.7)	179 (0.1)	145105
AR_DM1056	12077 (8.4)	131787 (91.5)	155 (0.1)	144019
AR_DM1088	12508 (8.7)	131840 (91.2)	161 (0.1)	144509
AR_KB635	13799 (9.6)	130496 (90.3)	181 (0.1)	144476
AR_KB652	13702 (9.4)	131878 (90.5)	186 (0.1)	145766
AR_KB754	12714 (8.9)	130679 (91.0)	179 (0.1)	143572
AR_KB819	12613 (8.8)	130130 (91.1)	169 (0.1)	142912
AR_KB820	11859 (8.2)	131991 (91.7)	152 (0.1)	144002
AR_KB827	13344 (9.2)	130830 (90.6)	187 (0.1)	144361
AR_KB945	12472 (8.6)	132022 (91.3)	153 (0.1)	144647
AR_MSH51	13891 (9.6)	131334 (90.3)	205 (0.1)	145430
AR_MSH126	14668 (9.9)	133024 (89.9)	236 (0.2)	147928
CH_JR20	22846 (7.7)	274352 (92.2)	429 (0.1)	297627
CH_JR198	22357 (7.6)	272449 (92.3)	417 (0.1)	295223
CH_JR272	22650 (7.7)	270334 (92.2)	361 (0.1)	293345
CH_JR356	21754 (7.7)	259309 (92.2)	306 (0.1)	281369
CH_JR377	22064 (7.5)	270738 (92.3)	394 (0.1)	293196
CH_KB888	21488 (7.4)	266728 (92.4)	388 (0.1)	288604
CH_MSH202	22843 (7.7)	274812 (92.2)	403 (0.1)	298058
PP_BdA1134-13	29089 (10.5)	246987 (89.3)	415 (0.2)	276491
PP_BdA1137-10	31345 (10.9)	255458 (88.9)	574 (0.2)	287377
PP_DM1038	22436 (7.4)	280484 (92.5)	394 (0.1)	303314
PP_DM1049	22814 (7.5)	280772 (92.4)	431 (0.1)	304017
PP_DM1054	21438 (7.1)	278961 (92.7)	393 (0.1)	300792
PP_DM1065	23950 (7.8)	282607 (92.0)	473 (0.2)	307030
PP_DM1081	24819 (8.0)	283619 (91.8)	535 (0.2)	308973
PP_DM1084	25483 (8.3)	281507 (91.5)	505 (0.2)	307495
PP_JR83	20670 (6.9)	278377 (93.0)	356 (0.1)	299403
ST_JR138	15760 (7.9)	183717 (92.0)	240 (0.1)	199717
ST_JR158	16684 (8.3)	183057 (91.5)	238 (0.1)	199979
ST_JR209	16315 (8.2)	181353 (91.6)	251 (0.1)	197919
ST_JR72	17677 (8.8)	182920 (91)	325 (0.2)	200922

ST_JR84	15488 (7.8)	184040 (92.1)	242 (0.1)	199770
ST_JR91	15869 (8.0)	182346 (91.9)	235 (0.1)	198450
ST_MSH177	17261 (8.6)	184169 (91.3)	233 (0.1)	201663
ST_MSH217	16634 (8.2)	185374 (91.7)	238 (0.1)	202246
TL_MA1959	29569 (10.4)	254859 (89.5)	409 (0.1)	284837
TL_MSH130	25155 (8.0)	289724 (91.9)	390 (0.1)	315269
TL_SCI_12-1	23987 (7.5)	293739 (92.3)	440 (0.1)	318166
TL_SPE123_2-3	23954 (7.5)	296292 (92.4)	398 (0.1)	320644
TL_SPE123_5-1	22076 (6.9)	296615 (93.0)	414 (0.1)	319105
TL_SPE123_6-3	27268 (8.4)	297866 (91.5)	510 (0.2)	325644
TL_SPE123_7-1	27355 (8.4)	297842 (91.4)	535 (0.2)	325732
TL_SPE123_8-1	24242 (7.6)	295852 (92.3)	428 (0.1)	320522

Het 0/1, Site called as heterozygous with the reference base and an alternative base; Hom 1/1, Site called as a SNP difference from the reference base; Het 1/2, Site called as heterozygous with two alternative bases.
