# Supplementary material for: Evidence for Stabilizing Selection on Codon Usage in Chromosomal Rearrangements of Drosophila pseudoobscura
Source: G3 (Bethesda). 2014 Oct 17;4(12):2433–49. doi: 10.1534/g3.114.014860 (PMC4267939; doi:10.1534/g3.114.014860)
Supplement: Supporting Information [file supp_g3.114.014860_TableS3.pdf]

Table S3   99% confidence intervals of the mean coverage

Strain	Location	Mean	Median	Mode	Cov_99.5%	Max
AR_DM1005	Davis Mts, TX	59.5	60	61	113	7996
AR_DM1015	Davis Mts, TX	46.4	47	47	94	5299
AR_DM1050	Davis Mts, TX	42.8	43	43	82	3661
AR_DM1056	Davis Mts, TX	31.5	32	32	62	3004
AR_DM1088	Davis Mts, TX	38.6	39	39	78	3801
AR_KB635	Kaibab NF, AZ	40.4	41	41	77	2385
AR_KB652	Kaibab NF, AZ	50.0	51	51	97	4346
AR_KB754	Kaibab NF, AZ	39.5	40	40	77	2662
AR_KB819	Kaibab NF, AZ	34.6	35	35	66	3122
AR_KB820	Kaibab NF, AZ	32.6	33	33	63	2290
AR_KB827	Kaibab NF, AZ	54.7	55	56	107	7955
AR_KB945	Kaibab NF, AZ	40.1	40	41	78	2871
AR_MSH126	Mt. St. Helena, CA	47.3	48	48	92	3948
AR_MSH51	Mt. St. Helena, CA	44.9	45	47	89	7801
PP_BdA1134-13	BosqueDelApache, NM	34.1	36	40	90	1429
PP_BdA1137-10	BosqueDelApache, NM	46.1	49	54	120	2482
PP_DM1038	Davis Mts, TX	39.2	41	42	75	2267
PP_DM1049	Davis Mts, TX	43.7	45	47	84	3425
PP_DM1054	Davis Mts, TX	38.5	40	41	73	3319
PP_DM1065	Davis Mts, TX	50.1	52	54	95	4109
PP_DM1081	Davis Mts, TX	57.1	59	62	101	4200
PP_DM1084	Davis Mts, TX	56.9	59	62	111	3531
PP_JR83	James Reserve, CA	35.0	36	38	68	1780
ST_JR138	James Reserve, CA	35.5	36	36	70	1772
ST_JR158	James Reserve, CA	45.0	45	46	88	3917
ST_JR209	James Reserve, CA	36.1	36	37	75	3425
ST_JR72	James Reserve, CA	55.4	56	57	107	4499
ST_JR84	James Reserve, CA	38.1	39	39	78	1667
ST_JR91	James Reserve, CA	34.1	34	35	64	2659
ST_MSH177	Mt. St. Helena, CA	43.1	44	44	84	2110
ST_MSH217	Mt. St. Helena, CA	44.8	46	46	90	1843
CH_JR198	James Reserve, CA	36.3	37	39	81	1947
CH_JR20	James Reserve, CA	37.3	38	40	75	2355
CH_JR272	James Reserve, CA	42.1	43	45	92	3202
CH_JR4	James Reserve, CA					
CH_JR356	James Reserve, CA	35.3	36	37	78	2205
CH_JR377	James Reserve, CA	34.0	34	35	72	4429

CH_KB888	Kaibab NF, AZ	41.6	43	45	86	3049
CH_MSH202	Mt. St. Helena, CA	38.4	40	41	80	1991
TL_MA1959	Mather, CA	34.0	35	39	94	1418
TL_MSH76	Mt. St. Helena, CA					
TL_MSH130	Mt. St. Helena, CA	39.2	40	41	84	5359
TL_SCI12-2	Santa Cruz Island, CA	47.5	49	51	99	3080
TL_SPE123_2-3	San Pablo Etla, Oaxaca, Mexico	42.8	43	45	97	7232
TL_SPE123_5-1	San Pablo Etla, Oaxaca, Mexico	39.6	41	43	82	2249
TL_SPE123_6-3	San Pablo Etla, Oaxaca, Mexico	51.2	53	56	112	7618
TL_SPE123_7-1	San Pablo Etla, Oaxaca, Mexico	53.5	55	58	128	7555
TL_SPE123_8-1	San Pablo Etla, Oaxaca, Mexico	39.6	41	43	85	3262
Dmir_SP138		14.6	14	17	86	2946
