# Supplementary material for: Evidence for Stabilizing Selection on Codon Usage in Chromosomal Rearrangements of Drosophila pseudoobscura
Source: G3 (Bethesda). 2014 Oct 17;4(12):2433–49. doi: 10.1534/g3.114.014860 (PMC4267939; doi:10.1534/g3.114.014860)
Supplement: Supporting Information [file supp_g3.114.014860_TableS4.pdf]

Table S4 Genes with significant high coverage

Gene	FBID_KEY	DROSOPHILA_ORTHOLOGS	LOC_MIN	LOC_MAX	LOC_STR
GA24632	FBgn0246022	-	1597752	1600079	1
GA15282	FBgn0075305	Rs1	2034368	2037130	1
GA24526	FBgn0245917	-	2038979	2039580	-1
GA24524	FBgn0245915	-	2039717	2040495	-1
GA24505	FBgn0245896	-	2576736	2580315	-1
GA24679	FBgn0083525	Or98a	3461540	3464987	1
GA24684	FBgn0246070	-	3505258	3506284	1
GA15652	FBgn0075669	NT5E-2	4828913	4831002	1
GA24437	FBgn0245834	-	4831115	4831843	-1
GA24421	FBgn0245818	-	5535422	5541561	-1
GA21477	FBgn0081464	-	6803237	6803734	-1
GA24775	FBgn0246161	tej	6858394	6860432	1
GA11906	FBgn0071955	CG12917	6878299	6879318	1
GA24778	FBgn0086700	Or46a	6879873	6882468	1
GA14766	FBgn0074793	CG18011	6882805	6886492	1
GA24369	FBgn0245766	-	7508444	7508899	-1
GA24821	FBgn0246206	-	8484241	8485145	1
GA10531	FBgn0070588	CG10737	9895870	9902715	1
GA14679	FBgn0074706	rdgBbeta	10628202	10630184	1
GA24283	FBgn02456	Cpr47Ef	1104570	11048905	-1

	80		6		
GA24280	FBgn0245677	CG14518, CG33725, CG33796	11175169	11175825	-1
GA21260	FBgn0081248	Cyp6a14	13261739	13263324	1
GA24236	FBgn0245635	CG13204	13533162	13536583	-1
GA15589	FBgn0075606	Gr47b	13540481	13541836	-1
GA15365	FBgn0075384	ptc	13682985	13696593	1
GA24978	FBgn0246361	CG7741	14053446	14055483	1
GA24214	FBgn0245613	CG33632, CG33912, CG33764	14218207	14218923	-1
GA12324	FBgn0072370	Gr58c	15426236	15427526	1
GA25040	FBgn0246423	CG30069	16304677	16311492	1
GA21181	FBgn0081169	Ih	17421113	17439461	1
GA24105	FBgn0245504	-	17703813	17707738	-1
GA30269	FBgn0263811	CG42678	17806002	17811813	-1
GA12384	FBgn0072430	CG13590, CG13589	18293905	18294537	1
GA30265	FBgn0263807	Strn-Mlck	18965505	18999327	1
