# Supplementary material for: Evidence for Stabilizing Selection on Codon Usage in Chromosomal Rearrangements of Drosophila pseudoobscura
Source: G3 (Bethesda). 2014 Oct 17;4(12):2433–49. doi: 10.1534/g3.114.014860 (PMC4267939; doi:10.1534/g3.114.014860)
Supplement: Supporting Information [file supp_g3.114.014860_TableS5.pdf]

Table S5 Tajima's *D* in *D. pseudoobscura* third chromosome gene arrangements.

Arrangement	Tajima's *D*
Arrowhead (AR)	-.7259
Pikes Peak (PP)	-.2548
Standard (ST)	-.2524
Tree Line (TL)	-.3517
Chiricahua (CH)	-.1317
