# Supplementary material for: Evidence for Stabilizing Selection on Codon Usage in Chromosomal Rearrangements of Drosophila pseudoobscura
Source: G3 (Bethesda). 2014 Oct 17;4(12):2433–49. doi: 10.1534/g3.114.014860 (PMC4267939; doi:10.1534/g3.114.014860)
Supplement: Supporting Information [file supp_g3.114.014860_TableS6.pdf]

Table S6 Cutoff values of *Fop* bins for each arrangement

Arrangement	Bin 1 (75-100%)	Bin 2 (50-75%)	Bin 3 (25-50%)	Bin 4 (0-25%)
Total	≥0.5962	0.5359-0.5962	0.4694-0.5359	≤0.4694
AR	≥0.5970	0.5367-0.5970	0.4710-0.5367	≤0.4710
ST	≥0.5976	0.5367-0.5976	0.4708-0.5367	≤0.4708
PP	≥0.5937	0.5342-0.5937	0.4684-0.5342	≤0.4684
TL	≥0.5953	0.5351-0.5953	0.4683-0.5351	≤0.4683
CH	≥0.5964	0.5354-0.5964	0.4703-0.5354	≤0.4703
