# Supplementary material for: Evidence for Stabilizing Selection on Codon Usage in Chromosomal Rearrangements of Drosophila pseudoobscura
Source: G3 (Bethesda). 2014 Oct 17;4(12):2433–49. doi: 10.1534/g3.114.014860 (PMC4267939; doi:10.1534/g3.114.014860)
Supplement: Supporting Information [file supp_g3.114.014860_TableS7.pdf]

Table S7 Cutoff values of recombination rate (ρ/bp) bins for each arrangement

Arrangement	Bin 1 (0-25%)	Bin 2 (25-50%)	Bin 3 (50-75%)	Bin 4 (75-100%)
Total	≤0.0232	0.0232-0.0405	0.0405-0.0655	≥0.0655
AR	≤0.0175	0.0175-0.0295	0.0295-0.0486	≥0.0486
ST	≤0.0044	0.0044-0.0100	0.0100-0.0193	≥0.0193
PP	≤0.0070	0.0070-0.0124	0.0124-0.0199	≥0.0199
TL	≤0.0108	0.0108-0.0199	0.0199-0.0296	≥0.0296
CH	≤0.0035	0.0035-0.0082	0.0082-0.0176	≥0.0176
