# Supplementary material for: Evidence for Stabilizing Selection on Codon Usage in Chromosomal Rearrangements of Drosophila pseudoobscura
Source: G3 (Bethesda). 2014 Oct 17;4(12):2433–49. doi: 10.1534/g3.114.014860 (PMC4267939; doi:10.1534/g3.114.014860)
Supplement: Supporting Information [file supp_g3.114.014860_TableS8.pdf]

Table S8 Cutoff values for genes grouped by percentiles of *ρ* spaced in 5% intervals.

Class	Percentile	ρ/bp
1	5%	≤0.0089
2	10%	0.0089-0.0125
3	15%	0.0125-0.0167
4	20%	0.0167-0.0203
5	25%	0.0203-0.0232
6	30%	0.0232-0.0266
7	35%	0.0266-0.0297
8	40%	0.0297-0.0332
9	45%	0.0332-0.0364
10	50%	0.0364-0.0405
11	55%	0.0405-0.0445
12	60%	0.0445-0.0484
13	65%	0.0484-0.0532
14	70%	0.0532-0.0576
15	75%	0.0576-0.0651
16	80%	0.0651-0.0722
17	85%	0.0722-0.0805
18	90%	0.0805-0.0948
19	95%	0.0948-0.1389
20	100%	≥0.1389
