# Supplementary material for: Evidence for Stabilizing Selection on Codon Usage in Chromosomal Rearrangements of Drosophila pseudoobscura
Source: G3 (Bethesda). 2014 Oct 17;4(12):2433–49. doi: 10.1534/g3.114.014860 (PMC4267939; doi:10.1534/g3.114.014860)
Supplement: Supporting Information [file supp_g3.114.014860_FileS1.pdf]

File S1

Supplementary Material

Relationship between recombination and codon bias

If a simple linear regression is performed on the average recombination rate (ρ/bp) for each gene and *Fop*, there is a weak yet statistically significant relationship (F=36.61, P=1.63x10^{-8}). The adjusted R-squared value is 0.01258.

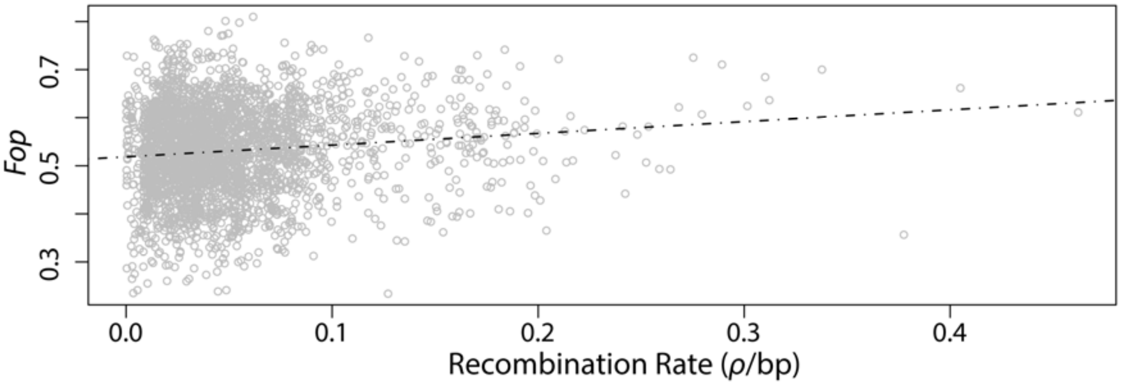

However, there are only 104 genes with average values of ρ greater than 0.2, which is likely influencing the result. Values of ρ do not appear to be normally distributed, which is an assumption of a simple linear regression. To create a more normal distribution of ρ values, a Box-Cox procedure was performed to determine the profile likelihood of the λ parameter for a power-transformation.

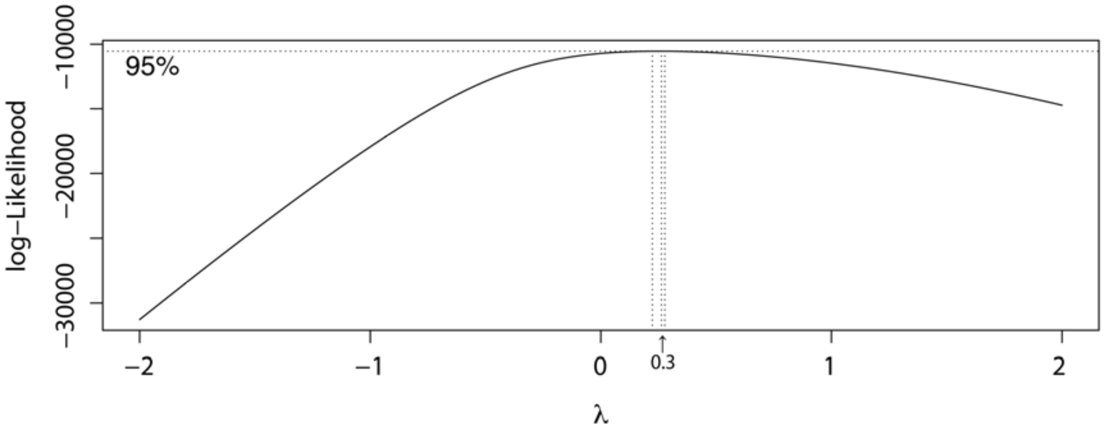

Values of ρ then underwent a power transformation with a λ parameter set to 0.3. For the transformed data, a significant relationship disappears (F=0.83, P=0.3624) and the correlation becomes even weaker (r=0.01249, R^2_{ADJ}=0.01214).

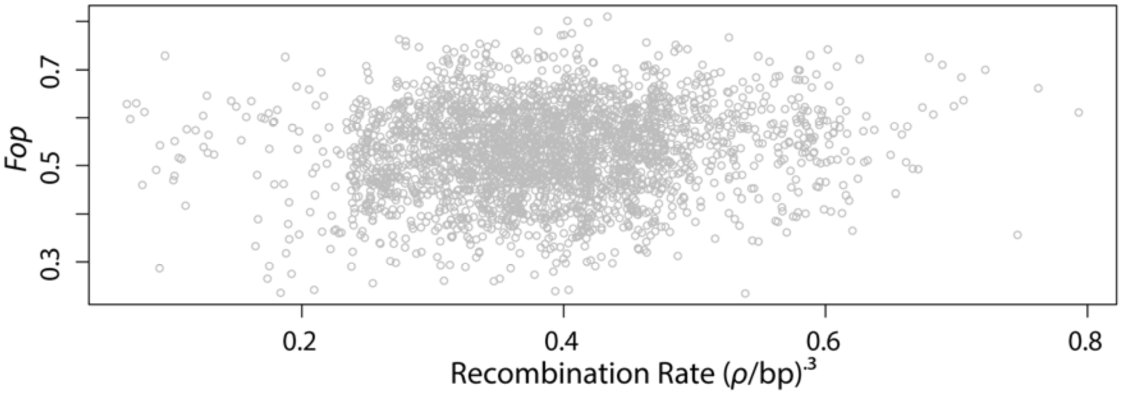

Fop
0.3
0.5
0.7
0.2
0.4
0.6
0.8
Recombination Rate (ρ/bp)^.3
